# Risk factors for drug-induced liver injury in tuberculosis patients: a meta-analysis and systematic review

**DOI:** 10.3389/fmed.2026.1834524

**Published:** 2026-04-29

**Authors:** Xiaoli Liu, Chunmei Liu, Rong Yao, Bin Wan, Li Fu, Xiaoyi Yang, Yan Zhang, Jinxia Du, Xiang Long

**Affiliations:** 1 The Fourth Department of Tuberculosis, Public Health Clinical Center of ChengDu, Chengdu, Sichuan, China; 2 The Department of Nursing, Public Health Clinical Center of ChengDu, Chengdu, Sichuan, China; 3 The Second Department of Tuberculosis, Public Health Clinical Center of ChengDu, Chengdu, Sichuan, China

**Keywords:** drug-induced liver injury, meta-analysis, risk factors, systematic review, tuberculosis

## Abstract

**Objective:**

A meta-analysis and systematic review of risk factors for drug-induced liver injury in tuberculosis patients.

**Methods:**

Computerized searches were conducted in PubMed, Embase, Web of Science, and the Cochrane Library databases to identify studies investigating risk factors for drug-induced liver injury in tuberculosis patients. The search period spanned from the inception of each database to January 2026. Two researchers independently screened studies, extracted data, and assessed risk of bias in included studies. Meta-analysis was performed using Stata 15.0 software.

**Results:**

A total of 41 studies involving 34,251 subjects were included. Meta-analysis revealed that female gender [OR = 1.35, 95% CI (1.05, 1.73)], alcohol consumption [OR = 2.07, 95% CI (1.57, 2.72)], extrapulmonary tuberculosis [OR = 2.01, 95% CI (1.66, 2.44)], disseminated TB [OR = 1.74, 95% CI (1.18, 2.59)], albumin <35 g/L [OR = 2.16, 95% CI (1.52, 3.06)], Malnutrition [OR = 3.32, 95% CI (1.52, 7.24)], HIV [OR = 1.80, 95% CI (1.09, 2.99)], HCV [OR = 2.17, 95% CI (1.10, 4.29)], hepatotoxic drugs [OR = 2.65, 95% CI (1.58, 4.43)] may constitute risk factors for drug-induced liver injury in tuberculosis patients.

**Conclusion:**

Female gender, alcohol consumption, extrapulmonary tuberculosis, disseminated tuberculosis, serum albumin levels below 35 g/L, malnutrition, HIV infection, HBsAg positivity, HCV infection, and hepatotoxic drugs may constitute risk factors for drug-induced liver injury in tuberculosis patients. Given limitations in the number and quality of included studies, these findings require validation through further high-quality research.

**Systematic review registration:**

This study has been registered on the PROSPERO platform, registration number: CRD420261302758.

## 1 Introduction

Tuberculosis is a chronic infectious disease transmitted through the air and remains the leading cause of death attributable to a single infectious agent, posing a formidable challenge to global public health. According to the World Health Organisation’s Global Tuberculosis Report 2025 (1), approximately 10.7 million people worldwide contracted tuberculosis, with 1.23 million deaths attributable to the disease – a death toll twice that of HIV/AIDS. Currently, anti-tuberculosis treatment employs multi-drug combination chemotherapy regimens (2). For instance, the WHO-recommended first-line short-course chemotherapy regimen comprises a quadruple combination of rifampicin, isoniazid, pyrazinamide, and ethambutol (3). Treatment protocols may be adjusted according to patient age, thereby ensuring a treatment success rate exceeding 85% (4).

However, the long-term combined use of multiple drugs inevitably leads to drug-related adverse reactions, among which drug-induced liver injury (DILI) stands as one of the most common side effects in tuberculosis treatment (5). Globally, studies reporting varying definitions of DILI, study populations, and treatment regimens indicate an overall incidence of anti-tuberculosis DILI ranging from 2.55 to 36.75% (6). Approximately 10 to 25% of patients are compelled to discontinue anti-tuberculosis therapy (7), with this complication tripling the risk of treatment failure and recurrence in tuberculosis patients (8), and in the most severe cases, it can be fatal (9), imposing a significant disease burden on tuberculosis patients. Consequently, the early identification of risk factors for DILI is a critical step in ensuring the safety of tuberculosis treatment.

To date, numerous studies have reported risk factors for DILI in tuberculosis patients, though findings vary across investigations. For instance, research has shown that being female is an independent risk factor for DILI (10), whereas other studies have shown that men are at a higher risk of developing DILI (11, 12). Some studies suggest that HIV increases the risk of drug-induced liver injury in patients with tuberculosis (13), although other studies have found no association between HIV infection and drug-induced liver injury in such patients (14).

Previously, a systematic review of risk factors for DILI in tuberculosis patients was limited to the Indian population (15), may not comprehensively capture risk variations across a global context. In recent years, with the publication of large-scale cohort studies and high-quality literature, the conclusions of previous reviews urgently require updating and integration. In light of this, the present study employs meta-analysis to systematically evaluate risk factors for DILI in tuberculosis patients, aiming to provide early warning for the prevention and management of its occurrence and progression.

## 2 Materials and methods

This study has been registered on the PROSPERO platform, registration number: CRD420261302758.

### 2.1 Literature search

PubMed, Embase, Web of Science, and the Cochrane Library were searched to comprehensively retrieve publications from the inception of each database up to 31 January 2026. The search strategy employed a combination of subject headings and free-text terms: tuberculosis; drug-induced liver injury; risk factors. Two trained researchers independently conducted the searches, while employing reference tracing to minimize search bias.

### 2.2 Inclusion and exclusion criteria

Inclusion criteria: (1) Patients with bacteriologically or clinically confirmed tuberculosis (according to WHO tuberculosis diagnostic guidelines); (2) Studies explicitly reporting risk factors for drug-related liver injury in tuberculosis patients; (3) Study types included: cross-sectional studies, case–control studies, and cohort studies; (4) Outcome measures: risk factors for drug-related liver injury.

Exclusion criteria: (1) Reviews, systematic reviews, conference proceedings, and case studies; (2) Duplicate publications; (3) Incomplete or unavailable literature; (4) Studies with flawed design, poor quality, or statistical errors.

### 2.3 Data extraction

Using EndNote X9 software, two researchers independently screened literature, extracted data, and cross-checked findings based on inclusion and exclusion criteria by reviewing titles, abstracts, and full texts. This ensured accuracy and consistency. In cases of disagreement, consensus was reached through discussion between the two researchers or by consultation with a third researcher. Extracted content comprised: (1) Basic information: first author, publication year, study location, study design, number of participants, number of DILI, gender distribution, age, definition of DILI, Risk factors; (2) Outcome measures: risk factors.

### 2.4 Quality assessment

To ensure the quality of studies included, two researchers independently assessed the quality of each paper using the appropriate quality assessment method selected according to the document type. Disagreements were resolved through discussion or third-party assistance, ultimately reaching consensus. For cross-sectional studies, the Agency for Healthcare Research and Quality (AHRQ) (16), comprising 11 assessment items evaluated as “yes,” “no,” or “unclear” (scored 1, 0, or 0 points respectively), with a maximum score of 11 points. Studies scoring 0–3, 4–7, or 8–11 points were classified as low, moderate, or high quality, respectively. For cohort and case–control studies, the Newcastle-Ottawa Scale (NOS)was employed to assess the quality of included literature (17). This scale comprises eight evaluation items, yielding a total score ranging from 0 to 9 points. Studies scoring 0–3 points, 4–6 points, and 7–9 points were classified as low, moderate, and high quality, respectively. Low-quality literature was excluded from the meta-analysis.

### 2.5 Data analysis

This study employed Stata 15.0 software for statistical analysis, with the combined effect size of risk factors expressed as odds ratios (OR) with 95% confidence intervals (CI). Heterogeneity among included studies was quantitatively assessed using the I-squared statistic (I^2^). An I^2^ value of less than 50% was considered to indicate low heterogeneity and substantial homogeneity across studies, thus warranting data synthesis and analysis using a fixed-effects model. Conversely, when the I^2^ value was 50% or higher, significant statistical heterogeneity was deemed to be present across studies, necessitating the use of a random-effects model for meta-analysis. The sources of heterogeneity were explored through sensitivity analyses. Publication bias was assessed using funnel plots and Egger’s test. Where funnel plot asymmetry or Egger’s test results indicated publication bias (*p* < 0.05), trim-and-trim correction was applied. A *p*-value < 0.05 was considered statistically significant.

## 3 Results

### 3.1 Literature search results

**Figure 1:**
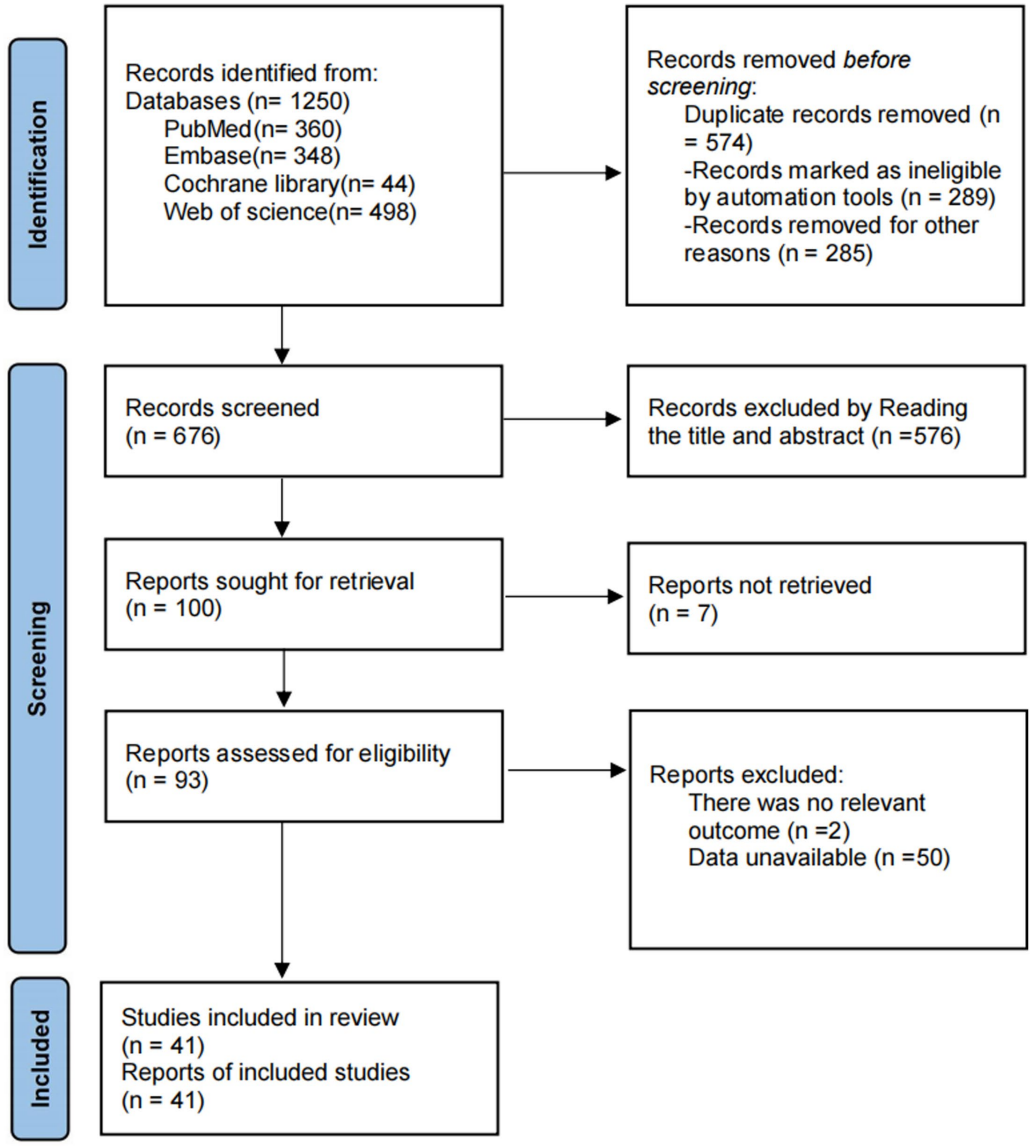
Literature screening process and results.

A total of 1,250 relevant publications were retrieved. After removing duplicates, 676 publications remained. Following review of titles and abstracts, 100 publications were selected. Based on inclusion and exclusion criteria, 41 publications were ultimately included after full-text review (6, 13, 14, 18–55). The literature screening process and results are presented in Figure 1.

### 3.2 Table of basic characteristics of included literature

This study included 41 published articles, comprising 2 cross-sectional studies, 31 cohort studies, and 8 case–control studies. These studies spanned 17 countries, encompassed all age groups, and involved a total of 34,251 patients, with a male-to-female ratio of 1:0.6189. The basic characteristics of the literature are presented in Table 1.

**Table 1:** The basic characteristics of the literature.

Author	Year	Country	Study design	Sample size	Number of DILI	Gender (M/F)	Age	Definition of DILI	**Risk factors**
Zhou Y	2025	China	Cohort study	482	18	259/223	0–14	Abnormal liver function was defined as any of ALT, AST, ALP, or TBil > ULN.	①⑥⑩
Zhou G F	2025	China	Cohort study	2,160	108	1408/752	≥**18**	(1) ALT ≥3 × ULN with compatible symptoms; (2) ALT ≥5 × ULN without symptoms; or (3) ALP ≥ 2 × ULN in the absence of bone pathology.	②③④⑥⑨
Pradhan	2025	Nepal	Cross-sectional study	78	12	45/33	≥**18**	ALT and AST ≥ 3 × ULN with hepatitis symptoms, or ≥5 × ULN without symptoms.	①③⑨⑱
Petros	2025	Ethiopia	Cohort study	219	35	109/110	≥18	ALT > 5 × ULN; or ALT > 3 × ULN with TBil > 2 × ULN; or ALP > 2 × ULN.	①②③⑭
Nyangwara	2025	South African	Case–control study	333	57	215/118	>18	ALT > 120 IU/L with symptoms, or ALT > 200 IU/L without symptoms.	③④⑤⑫⑭
Lim J	2023	Korea	Cohort study	684	52	431/253	/	(1) ALT ≥ 200 U/L, or ≥ 120 U/L with hepatitis symptoms; (2) AST ≥ 200 U/L, or ≥ 120 U/L with hepatitis symptoms; (3) TBil ≥ 3.0 mg/dL, or ≥ 1.5 mg/dL with hepatitis symptoms.	①②⑨
Ji S	2023	China	Cohort study	1979	83	/	/	Within 180 days post-dose, peak values met: ALT ≥ 3 × ULN; TBil ≥ 2 × ULN; or with AST, ALP, and TBil all elevated, any one ≥ 2 × ULN.	②④
Zhao P	2022	China	Case–control study	5,681	214	3342/2339	0.3–90	(1)ALT ≥3 × ULN / total bilirubin≥2 × ULN; or (2) total bilirubin≥2 × ULN or AST ≥ 2 × ULN or ALP ≥ 2 × ULN.	⑥⑩⑬
Xu N	2022	China	Cohort study	1,115	42	706/409	/	Concurrent elevation of ALT, AST, ALP, and TBil with any one ≥ 2 × ULN; or all elevated but within 2 × ULN.	②⑫⑱
Azis	2022	Indonesia	Cohort study	129	54	66/63	/	/	①④⑤⑪⑫⑳㉑
Zhong T	2021	China	Cohort study	757	287	494/263	/	(1)ALT≥80 U/L / total bilirubin≥40 μmol/L.	①⑱
Molla	2021	Ethiopia	Cohort study	216	17	98/118	≥**18**	ALT/AST > 3 × ULN with hepatitis symptoms and/or jaundice, or > 5 × ULN with or without symptoms.	②④
Mani	2021	India	Cohort study	393	43	241/152	/	(a) ALT/AST > 5 × ULN (asymptomatic); (b) ALT/AST > 3 × ULN with symptoms (anorexia, nausea, vomiting, jaundice); or (c) TBil > 1.5 mg/dL.	①②③⑧⑨⑬⑭
Liu Y H	2021	China	Cohort study	104	24	56/48	/	(1) Hepatocellular: ALT ≥ 3 × ULN, R ≥ 5; (2) Cholestatic: ALP ≥ 2 × ULN, R ≤ 2; (3) Mixed: ALT ≥ 3 × ULN, ALP ≥ 2 × ULN, 2 < R < 5. R = (ALT/ULN) ÷ (ALP/ULN).	①③⑲
Jiang F	2021	China	Cohort study	3,155	170	2019/1136	>16	(1) ALT ≥ 5 × ULN; (2) ALP ≥ 2 × ULN (with elevated 5′-nucleotidase or GGT, excluding bone pathology); or (3) ALT ≥ 3 × ULN with TBil > 2 × ULN.	②⑮㉑
Huang D	2021	China	Case–control study	129	78	95/34	/	ALT ≥ 5 × ULN; ALT ≥ 3 × ULN + TBil > 2 × ULN; or ALP ≥ 2 × ULN and RUCAM > 3.	①②⑨⑯
Khiewkhern	2020	Thai	Case–control study	327	21	209/118	/	AST/ALT > 3 × ULN symptomatic; AST/ALT > 5 × ULN asymptomatic; or TBil > 2 × ULN with normal AST/ALT.	②⑩⑭
Gezahegn	2020	Ethiopia	Cross-sectional study	188	26	106/82	≥**18**	ALT/AST ≥ 3 × ULN with hepatitis symptoms, or ≥ 5 × ULN without symptoms.	⑦⑨⑬⑭⑱
Gafar	2019	Indonesia	Cohort study	41	11	26/15	1–15	ALT or AST > 3 × ULN.	⑨⑱
Sun Q	2016	China	Cohort study	938	121	552/386	16–80	ALT/AST > 3 × ULN or TBil > 2 × ULN, after excluding other causes.	④⑨⑮
Lee	2016	Korea	Cohort study	299	29	254/45	>18	(1) ALT > 5 × ULN; (2) ALP > 2 × ULN; or (3) ALT > 3 × ULN with TBil > 2 × ULN.	⑬⑯⑰⑳
Kim	2016	Korea	Cohort study	379	52	132/247	/	ALT > 120 IU/L (symptomatic) or > 200 IU/L (asymptomatic).	⑯⑰⑳
Bright-Thomas	2016	UK	Cohort study	2070	63	1031/1039	/	AST/ALT > 5 × ULN, or TBil elevated.	①②
Abera	2016	Ethiopia	Cohort study	124	10	58/66	10–80	AST/ALT > 5 × ULN (asymptomatic); or > 3 × ULN with symptoms and TBil > 2 × ULN.	①②④⑥⑦
Trigo	2016	Brazil	Cohort study	100	41	79/21	≥**18**	ALT ≥ 2 × ULN (42 IU/L); if baseline ALT < 84 IU/L, requires ALT increase ≥ 2 × baseline level.	①②④⑤⑥⑧⑨⑭⑮⑰⑱⑲
Gaude	2015	India	Cohort study	3,900	150	2610/1290	/	(1) AST and/or ALT > 5 × ULN (50 IU/L); (2) TBil > 1.5 mg/dL; (3) Any AST/ALT elevation above baseline with anorexia, nausea, vomiting, jaundice; (4) No serologic evidence of HAV, HBV, or HCV infection.	①②④⑨⑪
Shu C C	2014	Taiwan	Cohort study	926	111	628/298	≥**18**	AST/ALT ≥ 3 × ULN symptomatic; or ≥ 5 × ULN (any presentation).	①⑭
Kato	2013	Japan	Cohort study	356	40	244/112	≥20	(1) ALT/AST > 3 × ULN (120 IU/L) + jaundice/hepatitis symptoms; (2) > 5 × ULN (200 IU/L); (3) TBil ≥ 2.0 mg/dL (any transaminase level).	⑱
Singla	2010	India	Case–control study	603	175	303/300	16–65	(1) AST/ALT > 5 × ULN (50 IU/L) once or > 3 × ULN (150 IU/L) × 3 consecutive; (2) TBil > 1.5 mg/dL; or (3) AST/ALT elevation with symptoms; plus (4) viral hepatitis excluded; and (5) Post-withdrawal improvement (TBil < 1 mg/dL, AST/ALT < 100 IU/L).	②⑨
Park	2010	Korea	Cohort study	107	18	81/26	/	(1) Any of: ALP/AST/ALT > 3 × ULN (normal baseline) or > 1.5 × baseline (abnormal baseline); or CTP > baseline; (2) Other causes excluded (e.g., HBV/HCV reactivation).	①⑱
de Castro	2010	Brazil	Cohort study	154	30	99/55	≥**18**	ALT > 2 × ULN (≥45 IU/L) per CIOMS; or for baseline ALT > 90 IU/L, ≥ 2 × baseline increase.	⑥⑮㉑
Marzuki	2008	Malaysia	Case–control study	473	46	/	/	At least 5 days after starting anti-TB drugs: AST/ALT ≥ 120 IU/L (normal < 40 IU/L) and/or TBil > 1.5 mg/dL (25 μmol/L) (normal < 1.5 mg/dL or < 25 μmol/L), with no other apparent cause.	⑥⑭
Makhlouf	2008	Egypt	Cohort study	100	15	44/56	/	(1) Post-withdrawal normalization of LFTs and symptom resolution; plus any of: (2) ALT/AST > ULN; (3) TBil > 5 mg/dL; (4) AST/ALT elevation with symptoms.	③⑬
Chang K C	2007	China	Case–control study	3,007	167	/	/	ALT ≥ 3 × ULN or TBil ≥ 2 × ULN.	④⑮
Senaratne	2006	Sri Lankan	Cohort study	783	74	565/218	/	symptoms (decreased appetite + nausea/vomiting) plus either SB > 1.1 mg/dL or ALT > 3 × ULN.	②
Subbalaxm	2020	India	Cohort study	200	28	113/87	18–65	AST ≥ 3 × ULN (symptomatic) or ≥ 5 × ULN (asymptomatic); and/or disproportionate TBil/ALP increase with symptoms.	①②④⑥⑦⑭
Mehra	2022	India	Cohort study	81	10	34/47	1–18	(1) ALT/AST > 3 × ULN (40 U/L) with hepatotoxicity symptoms; (2) ALT/AST > 5 × ULN regardless of symptoms; or (3) TBil > 1.5 mg/dL with or without symptoms.	㉑
Kumar	2025	India	Cohort study	140	38	63/77	/	(1) ALT ≥ 5 × ULN; (2) ALT ≥ 3 × ULN + TBil > 2 × ULN; (3) ALP ≥ 2 × ULN (particularly with elevated GGT).	⑧⑲
Pore	2014	India	Case–control study	893	56	630/263	≥**18**	ALT/AST > 3 × ULN with hepatitis symptoms and/or jaundice, or > 5 × ULN without symptoms.	①④
Khalili	2009	Iran	Cohort study	102	32	68/34	≥**18**	ALT or AST > 3 × ULN if symptomatic, or > 5 × ULN if asymptomatic.	①②④⑤⑪⑭⑯⑰⑱㉑
Akkahadsee	2024	Thailand	Cohort study	346	50	260/86	≥**18**	(1) ALT ≥ 5 × ULN; (2) ALP ≥ 2 × ULN (particularly with enzyme elevation, non-bone origin); (3) ALT ≥ 3 × ULN plus TBil ≥ 2 × ULN.	②④⑤⑩

① Female; ② ≥60 year; ③ BMI≥18.5; ④ Drinking; ⑤ Smoking; ⑥ Extrapulmonary TB; ⑦ Pulmonary; ⑧ Disseminated TB; ⑨ Albumin≥35 g/L; ⑩ Malnutrition; ⑪ DM; (12) Hypertension; (13) Liver Disease; ⑭ HIV; ⑮ HBsAg positive; ⑯ HBV; ⑰ HCV; ⑱ Hepatotoxic drugs; ⑲ Cholesterol; ⑳ AST >40 U/L; ㉑ ALT >40 IU/L.

### 3.3 Quality evaluation results

A quality assessment was conducted on the 41 included studies. Two cross-sectional studies scored 7 points using the AHRQ-developed scale. The NOS scale was applied to evaluate 31 cohort studies and 8 case–control studies, revealing that 4 studies scored 6 points and 35 studies scored ≥7 points. The quality of the included studies was thus classified as moderate to high. The results of the risk of bias assessment are presented in Table 2.

**Table 2:** Risk of bias assessment.

Cross-sectional											
Study	Whether the source of the information is clear	Whether exposed and non-exposed groups are listed	Whether a time was given to identify patients	If not, population derived, whether the subjects were consecutive	Whether the subjective factors of the evaluator cover up other aspects of the research object	Any assessment performed to ensure quality is described	The rationale for excluding any patients from the analysis was explained	Describe measures to evaluate and/or control for confounding factors	explain how missing data were handled in the analysis	Response rates and the completeness of data collection are summarized	If there is follow-up, identify the percentage of patients with expected incomplete data or follow-up results
Pradhan, 2025 (6)	Yes	Yes	Yes	NO	Unclear	Unclear	Yes	Yes	NO	Yes	Yes
Gezahegn, 2020 (14)	Yes	Yes	Yes	NO	Unclear	Unclear	Yes	Yes	NO	Yes	Yes

Cohort study									
Study	Representativeness of the exposed group	Selection of non-exposed groups	Determination of exposure factors	Identification of outcome indicators not yet to be observed at study entry	Comparability of exposed and unexposed groups considered in design and statistical analysis	design and statistical analysis	Adequacy of the study’s evaluation of the outcome	Adequacy of follow-up in exposed and unexposed groups	Total scores
Zhou Y, 2025 (55)	*	*	*	*	**	*	*	*	9
Zhou G F, 2025 (54)	*	*	*	*	**	*	*	*	9
Petros, 2025 (13)	*	*	*	*	**	*	/	/	7
Lim J, 2023 (35)	*	*	*	*	**	*	*	*	9
Ji S, 2023 (27)	*	*	*	*	*	*	*	/	7
Xu N, 2022 (51)	*	*	*	*	*	*	/	/	6
Azis, 2022 (20)	*	*	*	*	/	*	*	/	6
Zhong T, 2021 (53)	*	*	*	*	*	*	*	/	7
Molla, 2021 (41)	*	*	*	*	*	*	*	/	7
Mani, 2021 (38)	*	*	*	*	*	*	*	*	8
Liu Y H, 2021 (36)	*	*	*	*	*	*	*	*	8
Jiang F, 2021 (28)	*	*	*	*	**	*	*	*	9
Gafar, 2019 (24)	*	*	*	*	*	*	*	*	8
Sun Q, 2016 (49)	*	*	*	*	**	*	*	*	9
Lee, 2016 (34)	*	*	*	*	**	*	*	*	9
Kim, 2016 (32)	*	*	*	*	**	*	*	*	9
Bright-Thomas, 2016 (21)	*	/	*	*	/	*	*	*	6
Abera, 2016 (18)	*	*	*	*	*	*	*	*	8
Trigo, 2016 (50)	*	*	*	/	**	*	*	*	8
Gaude, 2016 (25)	*	*	*	*	**	*	*	/	8
Shu C C, 2014 (46)	*	*	*	*	**	*	*	/	8
Kato, 2013 (29)	*	*	*	*	**	*	*	/	8
Park, 2010 (43)	*	*	*	*	**	*	*	/	8
de Castro, 2010 (23)	*	*	*	*	**	*	*	*	9
Makhlouf, 2008 (37)	*	*	*	*	*	*	*	*	8
Senaratne, 2006 (45)	*	*	*	*	*	*	*	*	8
Subbalaxm, 2020 (48)	*	*	*	*	*	*	*	*	8
Mehra, 2022 (40)	*	*	*	*	*	*	*	/	7
Kumar, 2025 (33)	*	*	*	*	**	*	*	*	9
Khalili, 2009 (30)	*	/	*	*	/	*	*	*	6
Akkahadsee, 2024 (19)	*	*	*	*	**	*	*	*	9

Case control									
Study	Is the case definition adequate?	Representativeness of the cases	Determination of control group	Definition of controls	Comparability of cases and controls based on the design or analysis	Ascertainment of exposure	Same method of ascertainment for cases and controls	Non response	Total scores
Nyangwara, 2025 (42)	*	*	*	*	**	*	*	/	8
Zhao P, 2022 (52)	*	*	*	*	**	*	*	/	8
Huang D, 2021 (26)	*	*	*	*	**	*	*	*	9
Khiewkhern, 2020 (31)	*	*	*	*	**	*	*	/	8
Singla, 2010 (47)	*	*	*	*	**	*	/	/	7
Marzuki, 2008 (39)	*	*	*	*	**	/	*	/	7
Chang K C, 2007 (22)	*	*	*	*	**	*	*	/	8
Pore, 2014 (44)	*	/	*	*	**	*	*	*	8

### 3.4 Meta-analysis results

#### 3.4.1 Demographic characteristics

**Figure 2:**
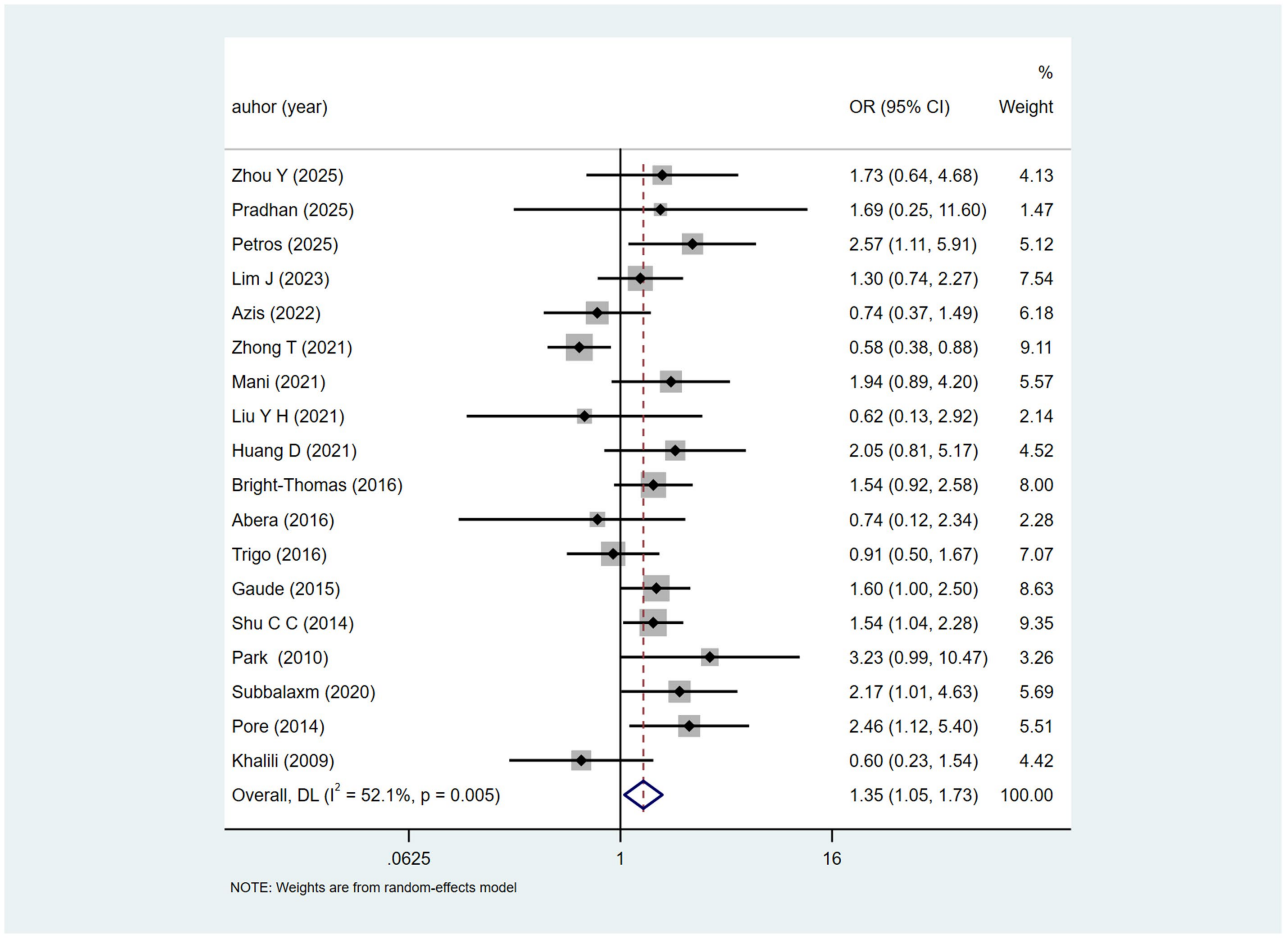
Female’s meta-analysis forest plot. The odds ratio (OR) with 95% confidence intervals (CI) were used to assess the association between Female and DILI [OR = 1.35, 95%CI (1.05, 1.73)].

The variable female was reported in 18 studies. Heterogeneity testing (I^2^ = 52.1%, *p* = 0.005). Analysed using a random-effects model, the results (Figure 2) suggest a potential association between female gender and DILI [OR = 1.35, 95% CI (1.05, 1.73)]. Sensitivity analyses indicated that the results were robust (Supplementary Figure S1).

#### 3.4.2 Behavioral factors

**Figure 3:**
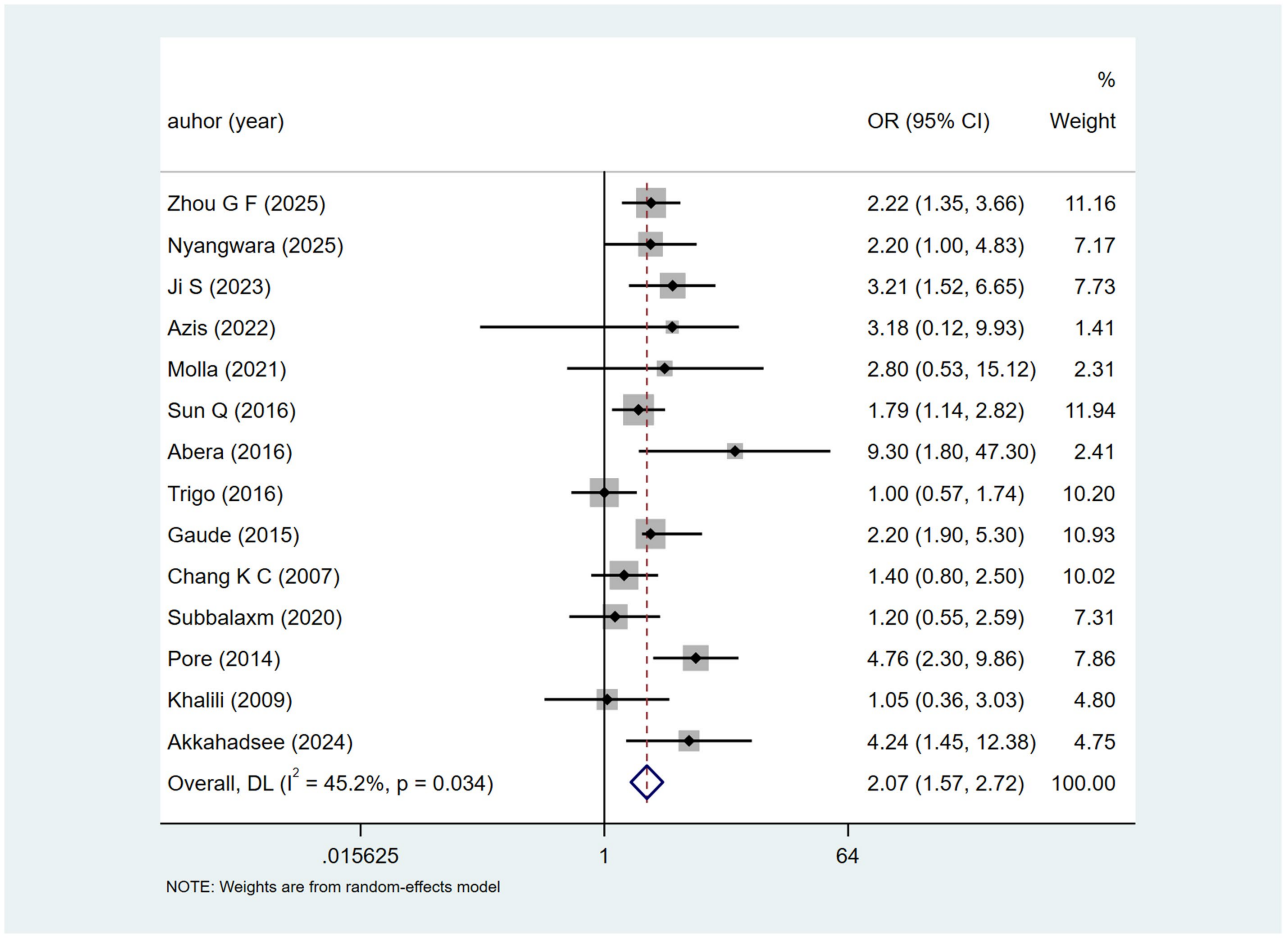
Drinking alcohol’s meta-analysis forest plot. The odds ratio (OR) with 95% confidence intervals (CI) were used to assess the association between Drinking alcohol and DILI [OR = 2.07, 95%CI (1.57, 2.72)].

Fourteen studies mentioned drinking alcohol. The heterogeneity test (I^2^ = 45.2%, *p* = 0.034) was conducted using a fixed-effects model. The analysis results (Figure 3) suggest that drinking alcohol may be associated with DILI [OR = 2.07, 95% CI (1.57, 2.72)].

#### 3.4.3 Disease/infection status

**Figure 4:**
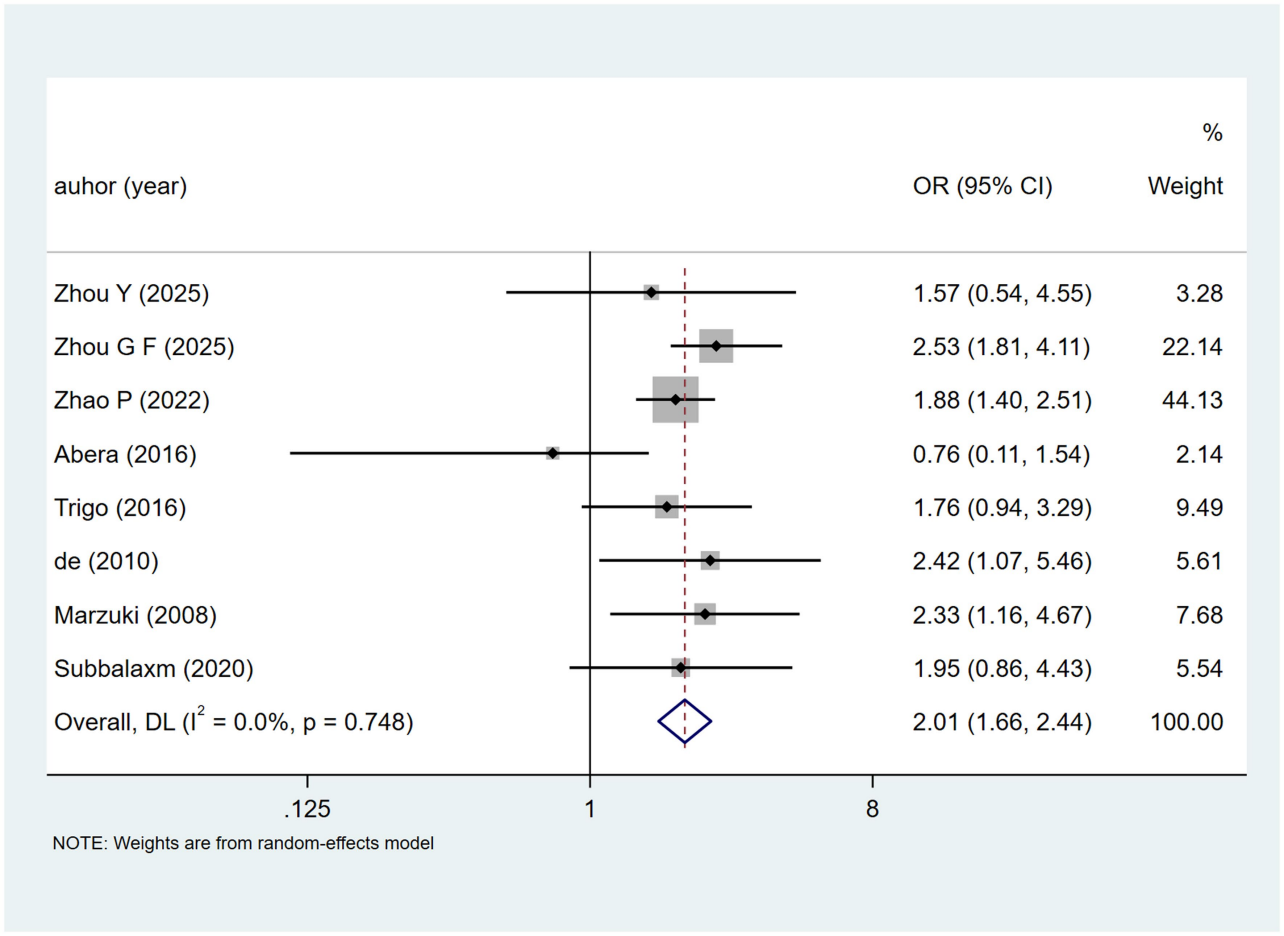
Extrapulmonary TB’s meta-analysis forest plot. The odds ratio (OR) with 95% confidence intervals (CI) were used to assess the association between Extrapulmonary TB and DILI [OR = 2.01, 95%CI (1.66, 2.44)].

In eight studies exploring extrapulmonary TB. heterogeneity was low (I^2^ = 0.0%, *p* = 0.748). A fixed-effects model (Figure 4) yielded [OR = 2.01, 95% CI (1.66, 2.44)], pointing to a possible link with DILI.

**Figure 5:**
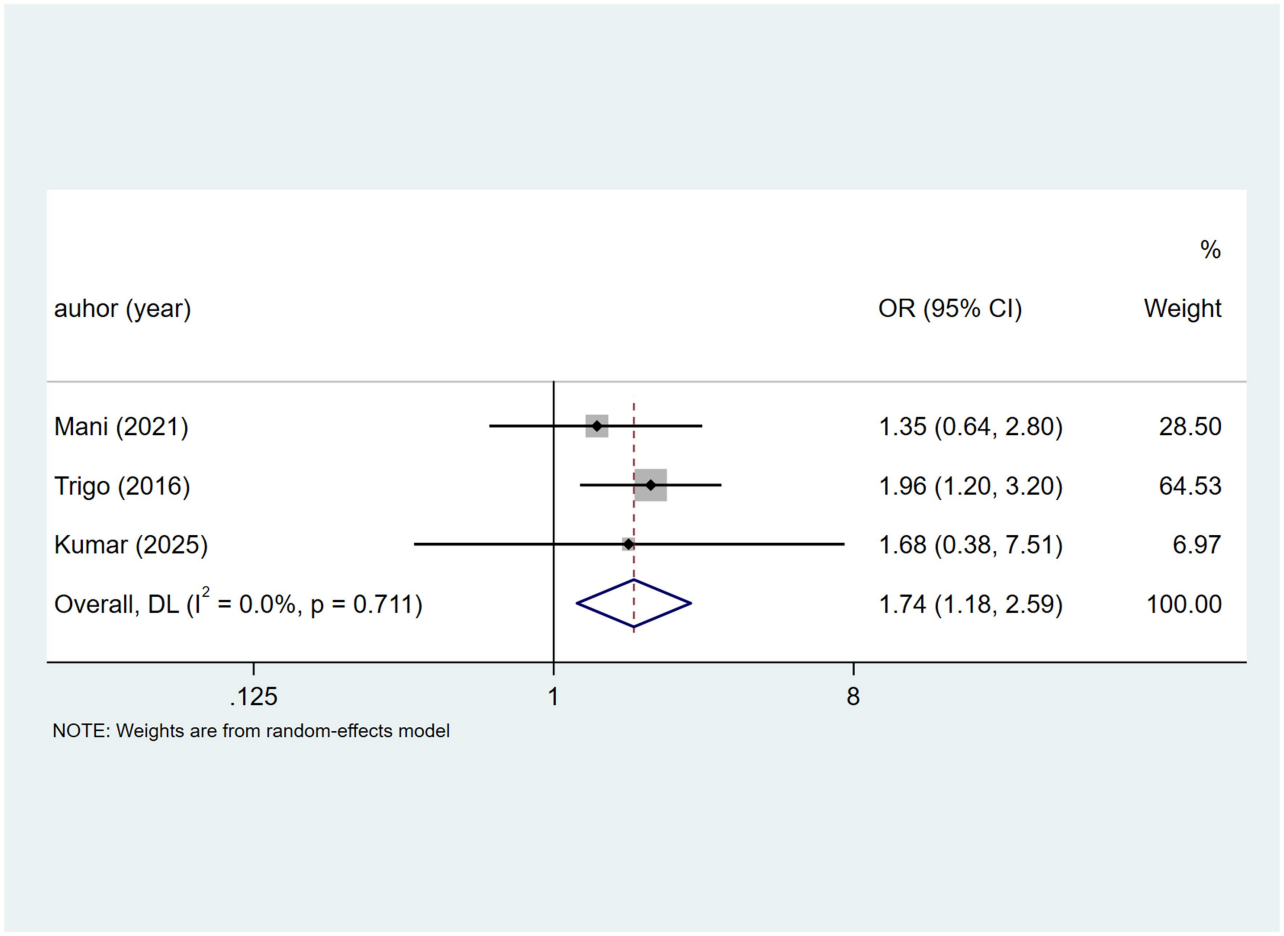
Disseminated TB’s meta-analysis forest plot. The odds ratio (OR) with 95% confidence intervals (CI) were used to assess the association between Disseminated TB and DILI [OR = 1.74, 95%CI (1.18, 2.59)].

Three studies examined disseminated TB. With no heterogeneity detected (I^2^ = 0.0%, *p* = 0.711). a fixed-effects analysis (Figure 5) suggesting an association with DILI [OR = 1.74, 95% CI (1.18, 2.59)].

**Figure 6:**
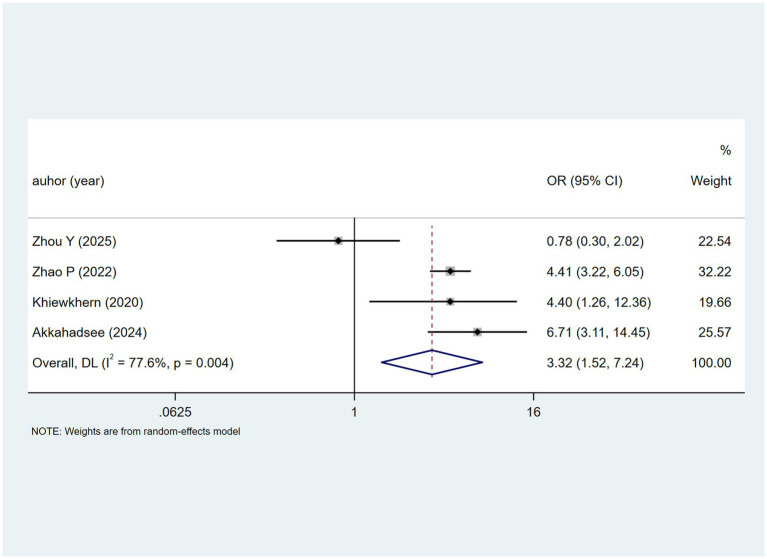
Malnutrition’s meta-analysis forest plot. The odds ratio (OR) with 95% confidence intervals (CI) were used to assess the association between Malnutrition and DILI [OR = 3.32, 95%CI (1.52, 7.24)].

Malnutrition was examined in four studies, with heterogeneity testing showing (I^2^ = 77.6%, *p* = 0.004). Analysed using a random-effects model, the results (Figure 6) suggest malnutrition may be associated with DILI [OR = 3.32, 95% CI (1.52, 7.24)]. Sensitivity analysis (Supplementary Figure S2) indicates the results are stable and not influenced by individual studies.

**Figure 7:**
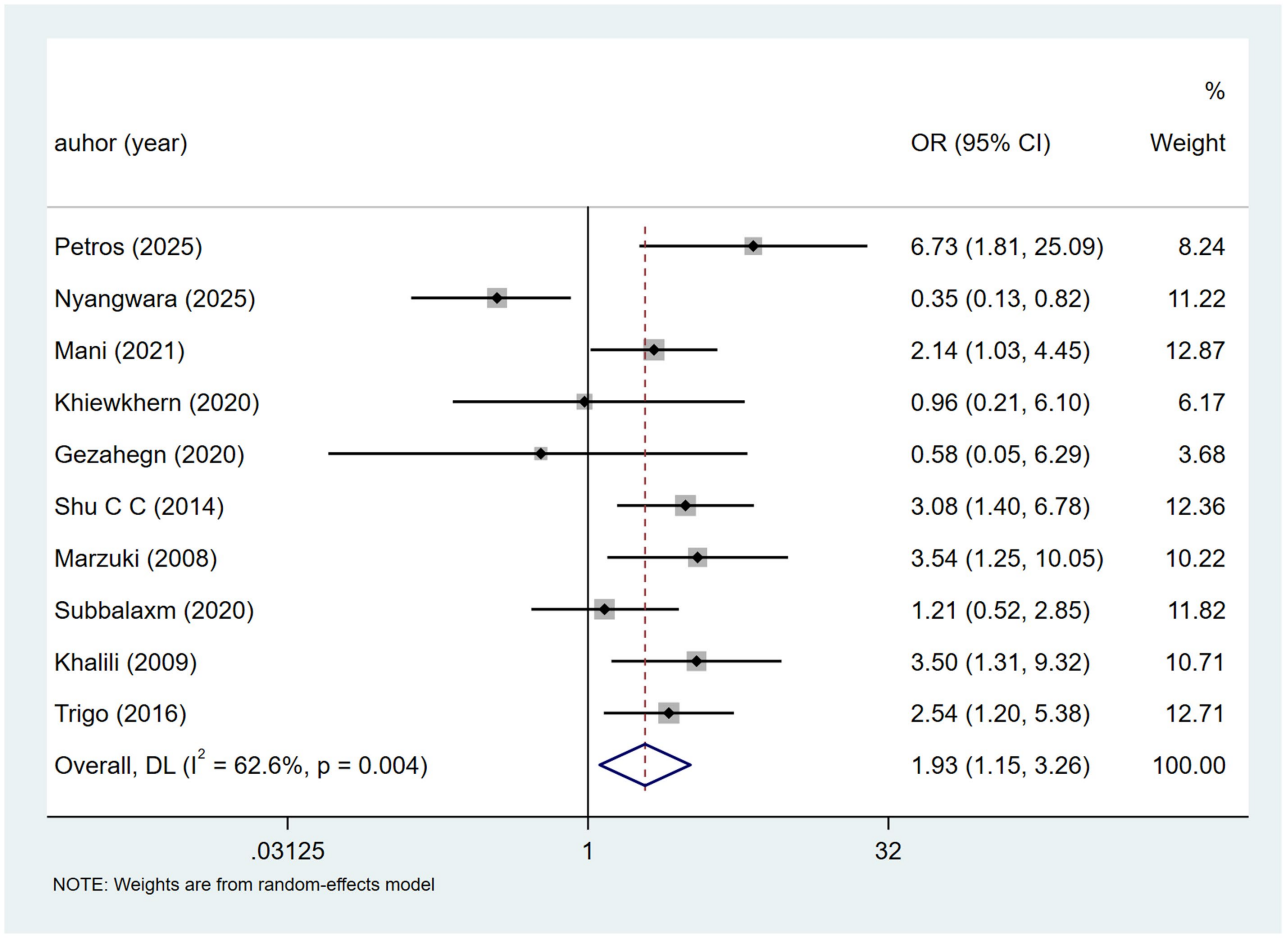
HIV’s meta-analysis forest plot. The odds ratio (OR) with 95% confidence intervals (CI) were used to assess the association between HIV and DILI [OR = 1.93, 95% CI (1.15, 3.26)].

HIV from ten studies showed heterogeneity (I^2^ = 62.6%, p = 0.004). A random-effects model (Figure 7) suggested a potential association between HIV and DILI [OR = 1.93, 95% CI (1.15, 3.26)]. Sensitivity analysis (Supplementary Figure S3) found no influence from any single study.

**Figure 8:**
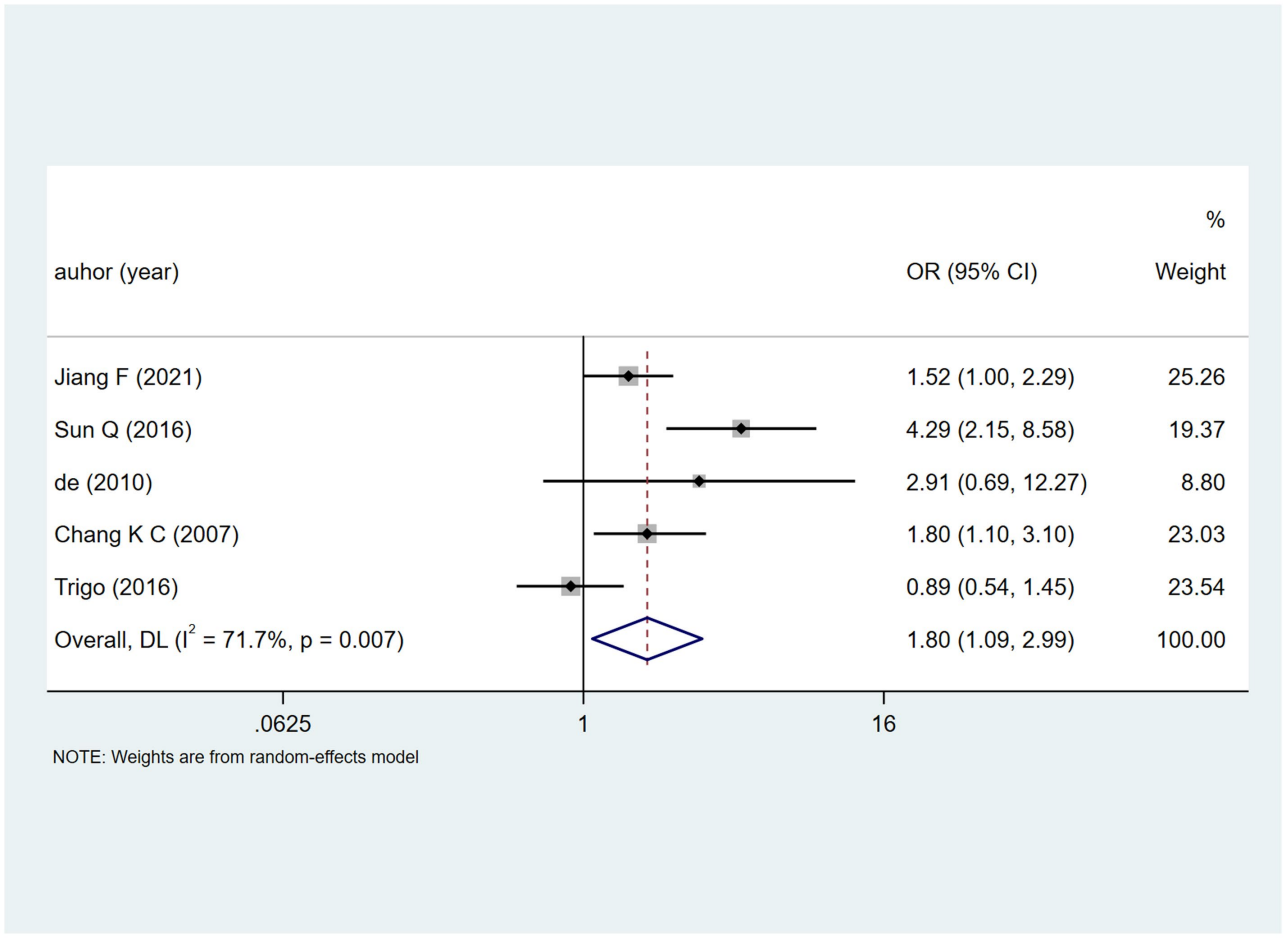
HBsAg positive’s meta-analysis forest plot. The odds ratio (OR) with 95% confidence intervals (CI) were used to assess the association between HBsAg positive and DILI [OR = 1.80, 95%CI (1.09, 2.99)].

HBsAg positive status across five studies yielded heterogeneity (I^2^ = 71.7%, *p* = 0.007). The random-effects model (Figure 8) gave [OR = 1.80, 95% CI (1.09, 2.99)], hinting at a possible link to DILI. Sensitivity analysis (Supplementary Figure S4) showed stable results unaffected by individual studies.

**Figure 9:**
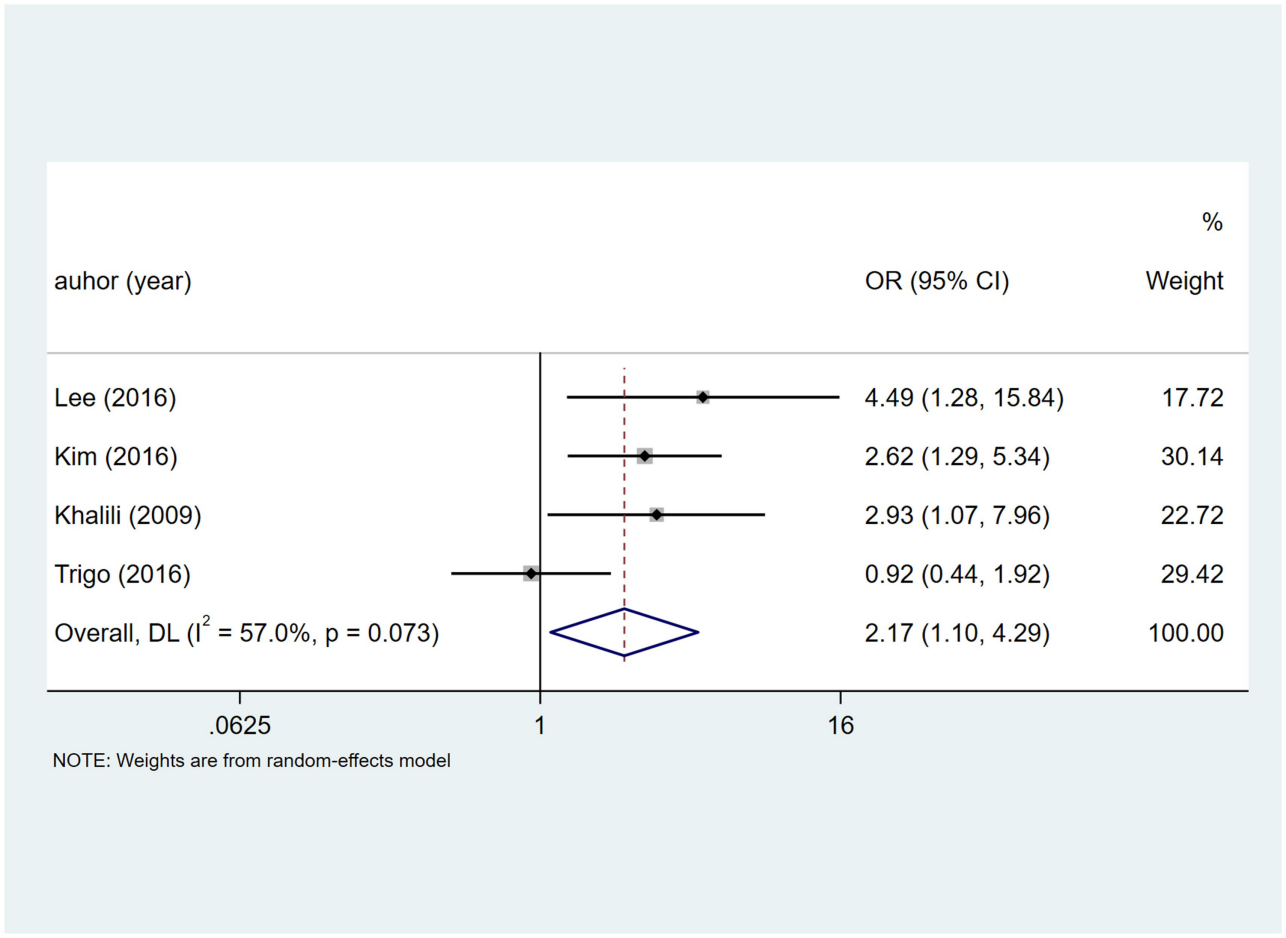
HCV’s meta-analysis forest plot. The odds ratio (OR) with 95% confidence intervals (CI) were used to assess the association between HCV and DILI [OR = 2.17, 95%CI (1.10, 4.29)].

Based on four studies, HCV had heterogeneity (I^2^ = 57.0%, *p* = 0.073). A random-effects model (Figure 9) indicated a potential DILI association [OR = 2.17, 95% CI (1.10, 4.29)], and sensitivity analysis (Supplementary Figure S5) indicated stability.

#### 3.4.4 Laboratory parameters

**Figure 10:**
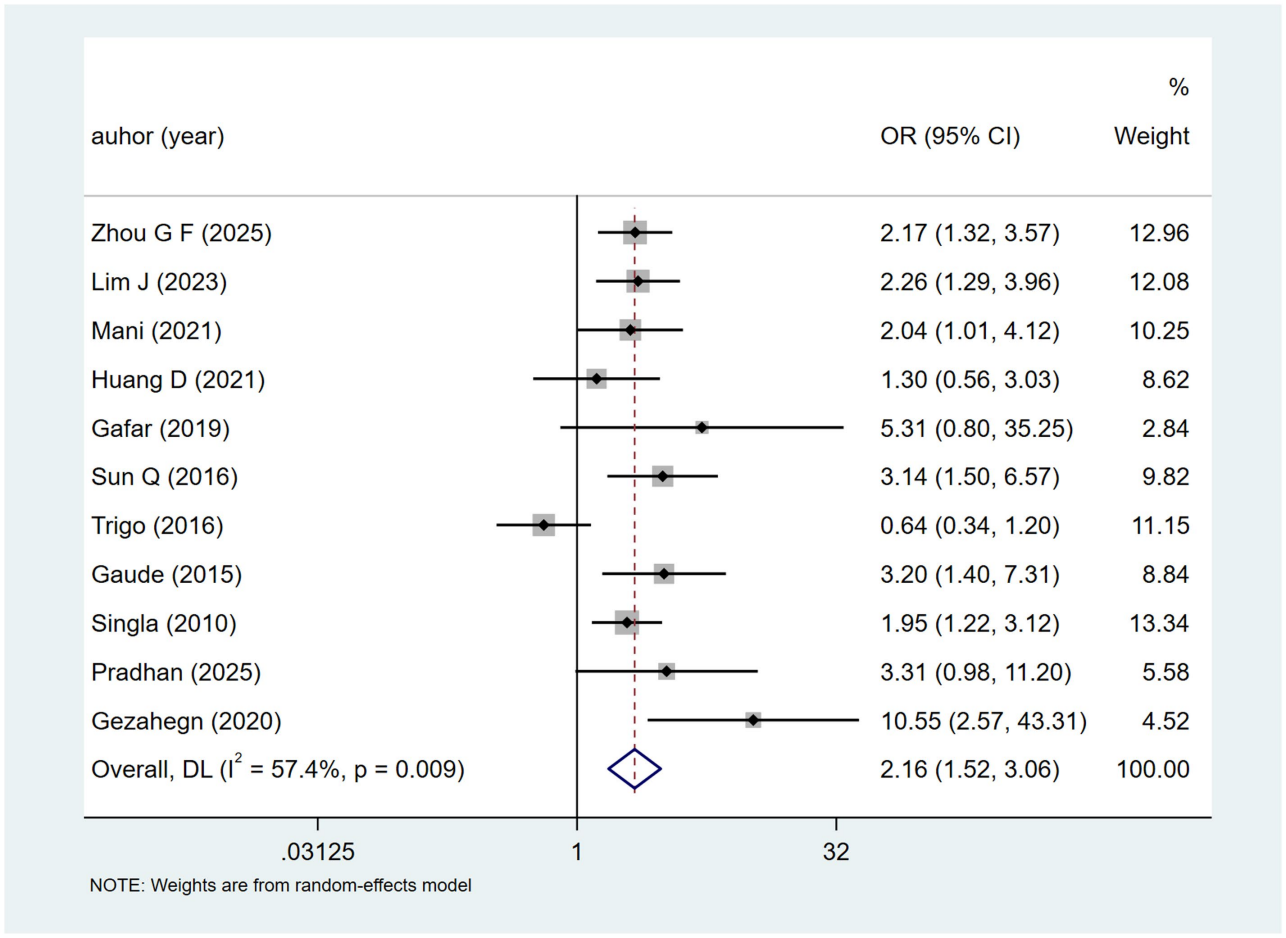
Albumin <35 g/L’s meta-analysis forest plot. The odds ratio (OR) with 95% confidence intervals (CI) were used to assess the association between Albumin <35 g/L and DILI [OR = 2.16, 95%CI (1.52, 3.06)].

Albumin <35 g/L from eleven studies had heterogeneity (I^2^ = 57.4%, *p* = 0.009). A random-effects model (Figure 10) suggested a potential association with DILI [OR = 2.16, 95% CI (1.52, 3.06)]. Sensitivity analysis (Supplementary Figure S6) indicated stability.

#### 3.4.5 Drug-related

**Figure 11:**
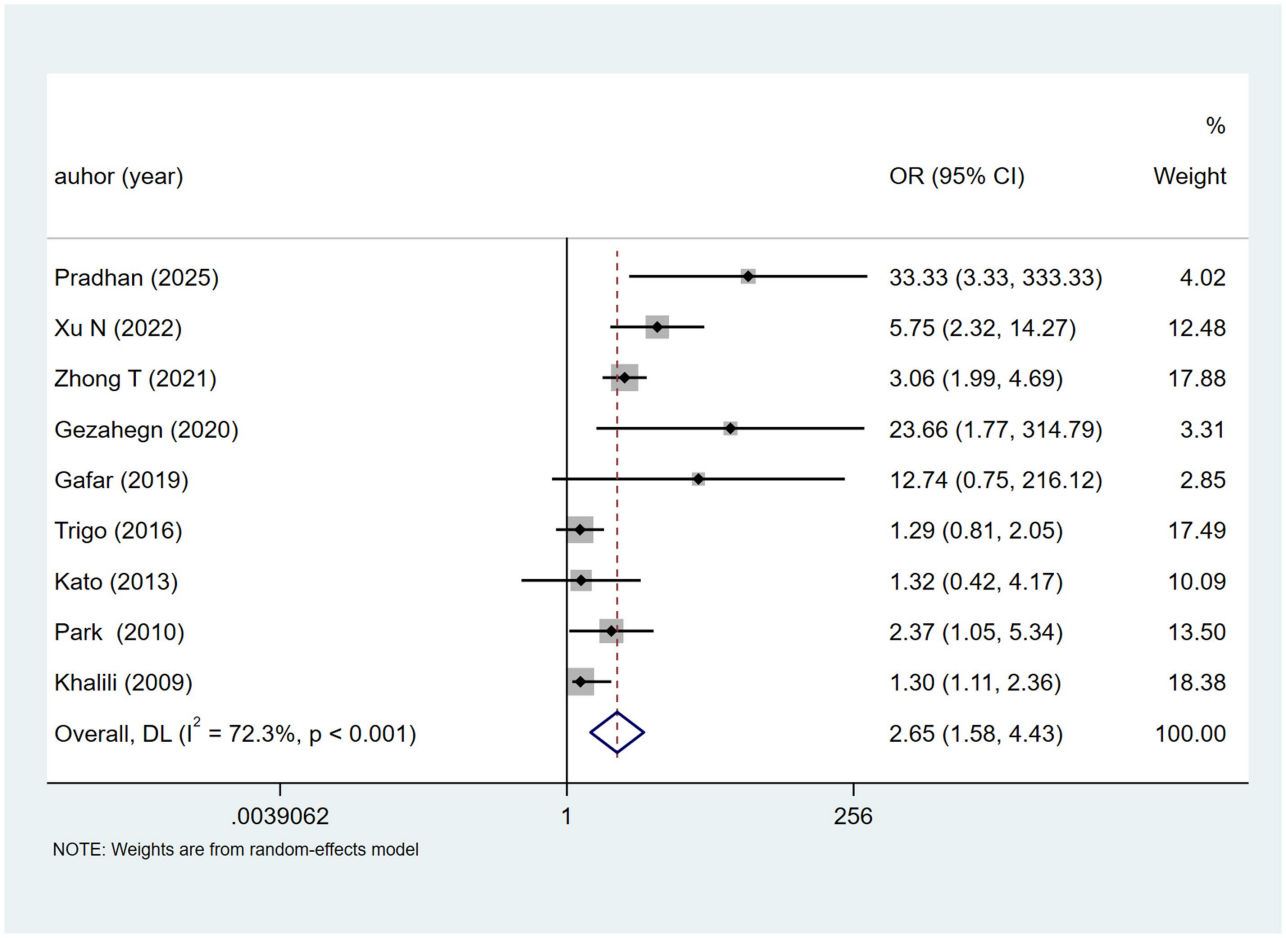
Hepatotoxic drugs’s meta-analysis forest plot. The odds ratio (OR) with 95% confidence intervals (CI) were used to assess the association between hepatotoxic drugs and DILI [OR = 2.65, 95%CI (1.58, 4.43)].

Hepatotoxic drugs was recorded in Nine studies. Heterogeneity testing (I^2^ = 72.3%, *p* < 0.001) was analysed using a random-effects model. Results (Figure 11) suggested a potential association with DILI [OR = 2.65, 95% CI (1.58, 4.43)]. Sensitivity analysis (Supplementary Figure S7) indicated stability.

### 3.5 Publication offset

Using Stata 15.0 statistical software to construct funnel plots, we examined whether publication bias was present based on the results. The funnel plots for the aforementioned findings were largely symmetrical (Supplementary Figures S8–S17).

## 4 Discussion

This study conducted a comprehensive literature search, ultimately including 41 studies from 15 countries involving 34,251 patients. The types of studies included were cross-sectional studies, cohort studies, and case–control studies. The majority of included studies were of moderate to high quality. Sensitivity analyses were conducted to explore the impact of different effect models and individual studies on the pooled effect size. Results indicated that female gender, alcohol consumption, extrapulmonary tuberculosis, disseminated tuberculosis, albumin levels <35 g/L, malnutrition, HIV infection, HBsAg positivity, HCV infection, and hepatotoxic drugs constituted risk factors for DILI in tuberculosis patients.

Regarding gender factors, the findings of this study suggest that women are more susceptible to DILI. Potential explanations include the influence of the unique cyclical variations in estrogen and progesterone levels on human immune function, alongside the role of sex hormones in regulating drug metabolism and transport, thereby affecting the host’s response to exogenous medications (56). Additionally, this susceptibility may stem from slower acetylation patterns and lower body mass index (BMI) (13).

Previous studies have confirmed that alcohol consumption constitutes a significant risk factor for DILI. Alcohol consumption induces the production of CYP2E1 and other CYP450 isoenzymes, thereby increasing the generation of reactive oxygen species and enhancing hepatotoxicity, particularly when used in conjunction with anti-tuberculosis drugs such as isoniazid and rifampicin (57). Fernández-Villar et al. found that chronic alcoholism increases the risk of DILI by 1.79-fold (58).

Multiple studies indicate that patients with extrapulmonary tuberculosis or disseminated tuberculosis exhibit a higher incidence of DILI. This may be attributable to the extended treatment duration or more intensive anti-tuberculosis regimens employed in such cases, coupled with the presence of heightened systemic inflammation. Furthermore, the disease course frequently involves the use of second-line drugs or adjunctive medications with hepatotoxic potential (59).

Research indicates that albumin levels below 35 g/L are significantly associated with the occurrence of DILI. Albumin reflects an individual’s overall health status, and its decline may be influenced by chronic liver disease, renal disease, severe infections, and inflammatory states. These pathological conditions reduce drug clearance rates, leading to drug concentrations exceeding threshold levels. This, in turn, may induce or exacerbate chronic inflammatory responses (60), thereby accelerating the progression of DILI.

Malnutrition constitutes a significant risk factor for DILI. Malnutrition may reduce the activity of drug-metabolizing enzymes, decrease the liver’s intrinsic clearance capacity, and weaken antioxidant defenses, leading to drug accumulation in the liver and subsequent hepatic damage. Research indicates that providing nutritional support to tuberculosis patients may enhance treatment tolerance and reduce the risk of hepatotoxicity (61).

Research indicates that the risk of DILI in HCV patients is approximately five times that of the general population, while the relative risk in HIV patients is four times higher. For those co-infected with HCV and HIV, the risk rises significantly to 14.4 times that of the general population (62). This may stem from immune dysfunction, which impairs the ability of HIV-positive individuals to respond efficiently to oxidative stress and metabolize drug products (63).

Research indicates that HBsAg-positive patients are more susceptible to DILI. Compared with HBsAg-negative patients, HBsAg-positive patients face a 4.29-fold increased risk of developing DILI (64). This finding suggests that during clinical medication, enhanced monitoring of liver function in HBsAg-positive patients is warranted to effectively prevent and reduce the occurrence of DILI.

Hepatotoxic drugs are high-risk factors for DILI. Anti-tuberculosis therapy relies on first-line drugs such as isoniazid, rifampicin, ethambutol, and pyrazinamide. However, these agents exhibit potent hepatotoxicity (65), with toxic effects that not only disrupt hepatic cell structure and function but also interfere with metabolic pathways and mitochondrial function (66). Prolonged combined use may consequently induce DILI.

This study identifies risk factors for DILI caused by anti-tuberculosis drugs, providing evidence-based guidance for clinical identification of high-risk populations. During anti-tuberculosis treatment, patients exhibiting risk factors may undergo more frequent liver function monitoring, optimized drug combinations and dose adjustments, or even prophylactic hepatoprotective measures. This facilitates a shift from reactive management to proactive prevention, thereby minimizing DILI incidence, ensuring smooth implementation of treatment regimens, and enhancing overall tuberculosis cure rates.

Limitations of this study: (1) The inclusion of studies did not encompass all high-burden tuberculosis countries, potentially affecting the comprehensiveness and representativeness of the results. (2) Variations in the definition of DILI across different studies may introduce heterogeneity. (3) The limited number of studies addressing certain risk factors may influence the findings. (4) The inclusion of multiple study designs beyond cohort studies resulted in considerable heterogeneity among the included research.

## 5 Conclusion

In summary, female gender, alcohol consumption, extrapulmonary tuberculosis, disseminated tuberculosis, serum albumin <35 g/L, malnutrition, HIV infection, HBsAg positivity, HCV infection, and hepatotoxic drugs may constitute risk factors for DILI in tuberculosis patients. In future research, it is recommended that a scoring system be developed to predict the risk of DILI in patients with tuberculosis. Such a scoring system would be of considerable practical value in regions with high prevalence of tuberculosis, as adverse events related to DILI frequently occur during anti-tuberculosis treatment, yet are often underestimated in clinical practice.

## Data Availability

The original contributions presented in the study are included in the article/Supplementary material, further inquiries can be directed to the corresponding author.
