# Supplementary material for: Risk factors for drug-induced liver injury in tuberculosis patients: a meta-analysis and systematic review
Source: Front Med (Lausanne). 2026 Apr 29;13:1834524. doi: 10.3389/fmed.2026.1834524 (PMC13167532; doi:10.3389/fmed.2026.1834524)
Supplement: Supplementary file 1 [file Data_Sheet_1.doc]

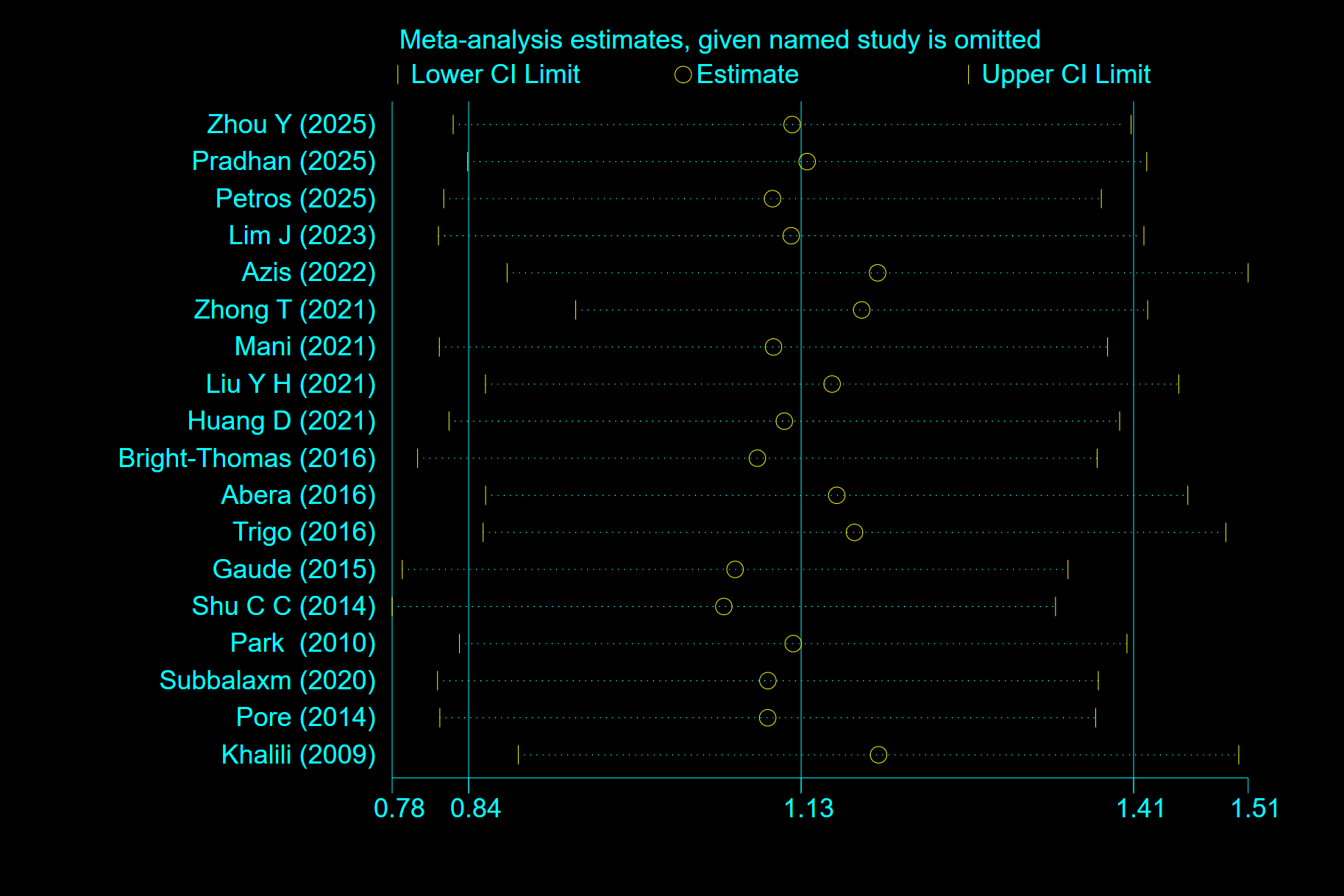


figure S1


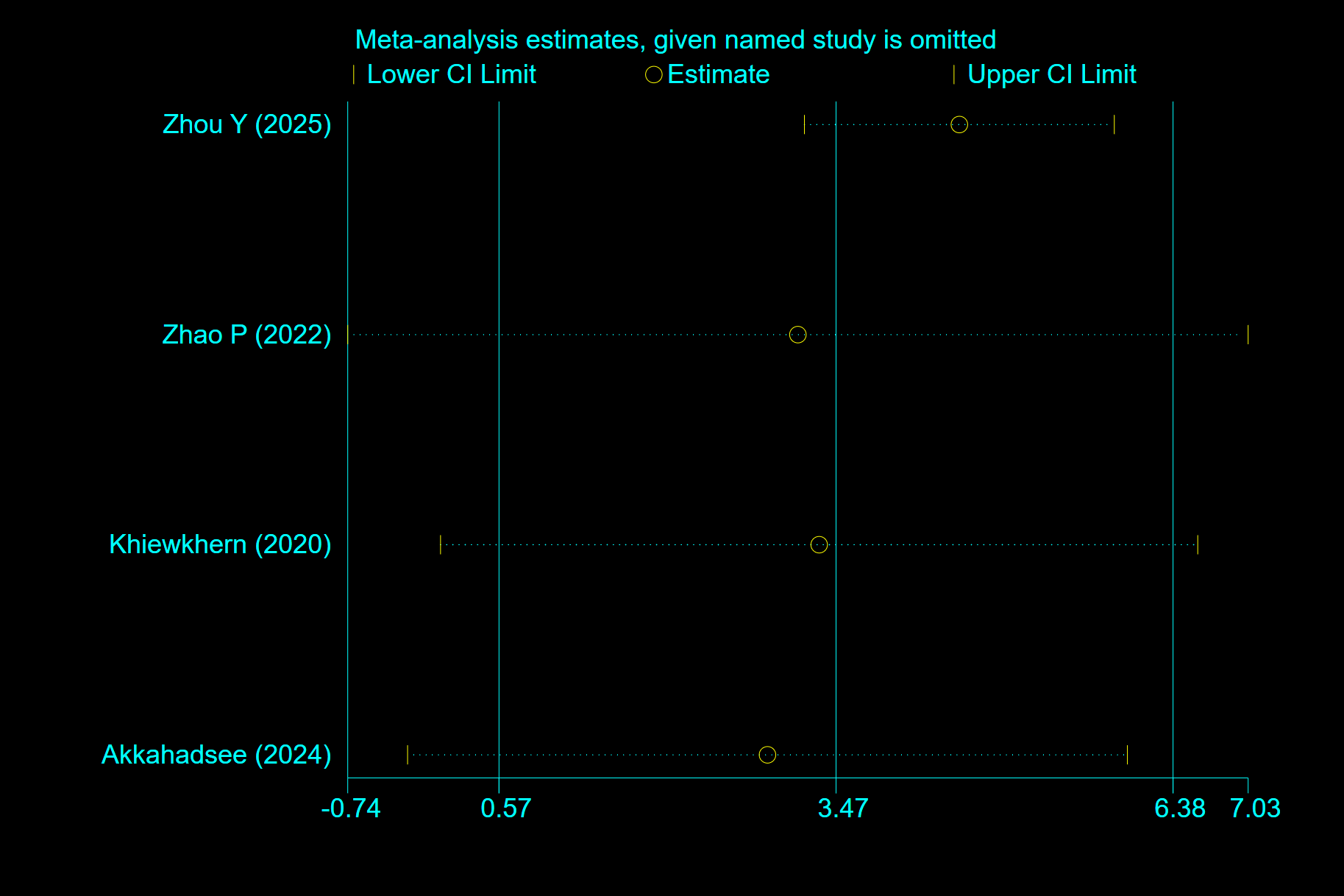
figure S2


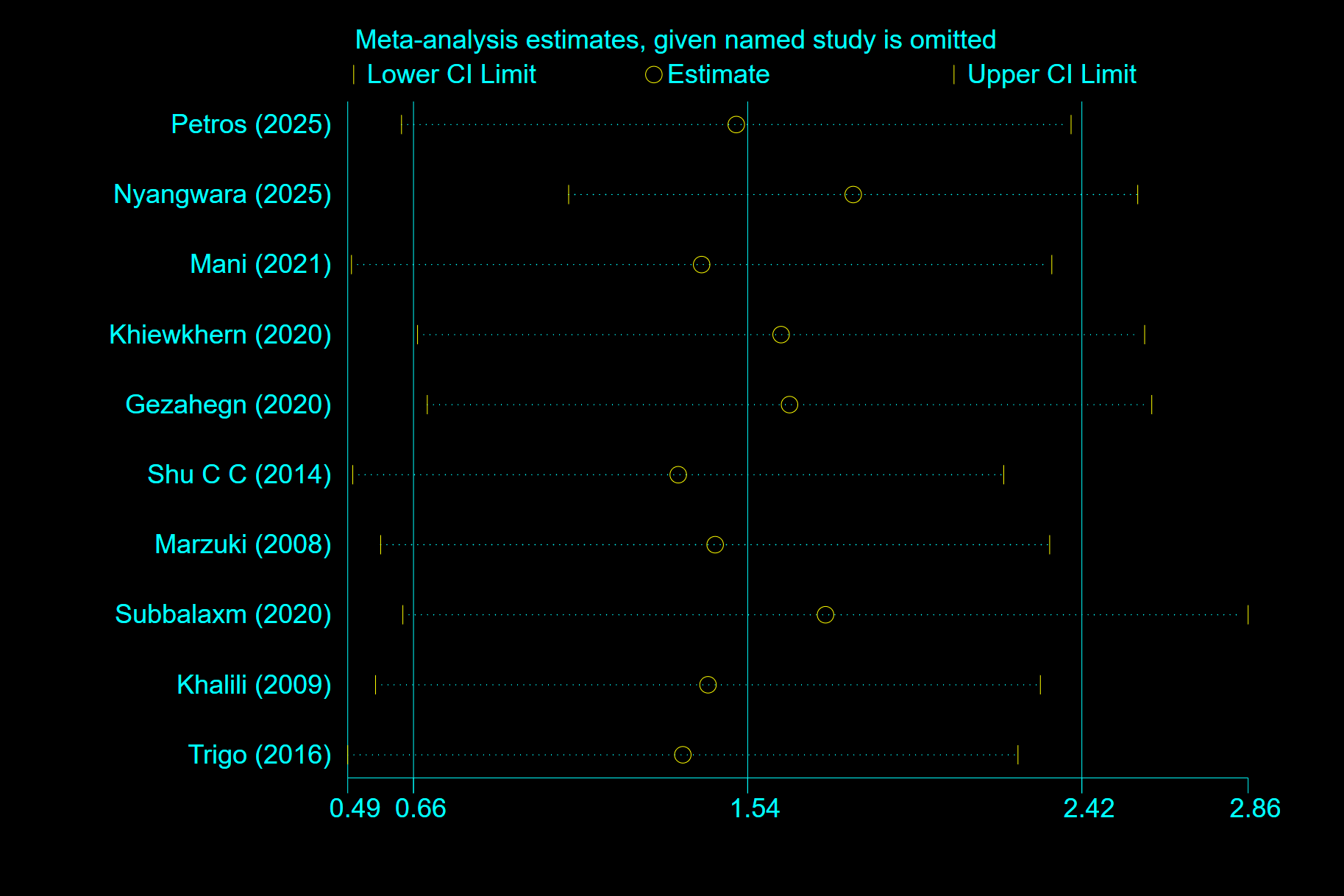
figure S3


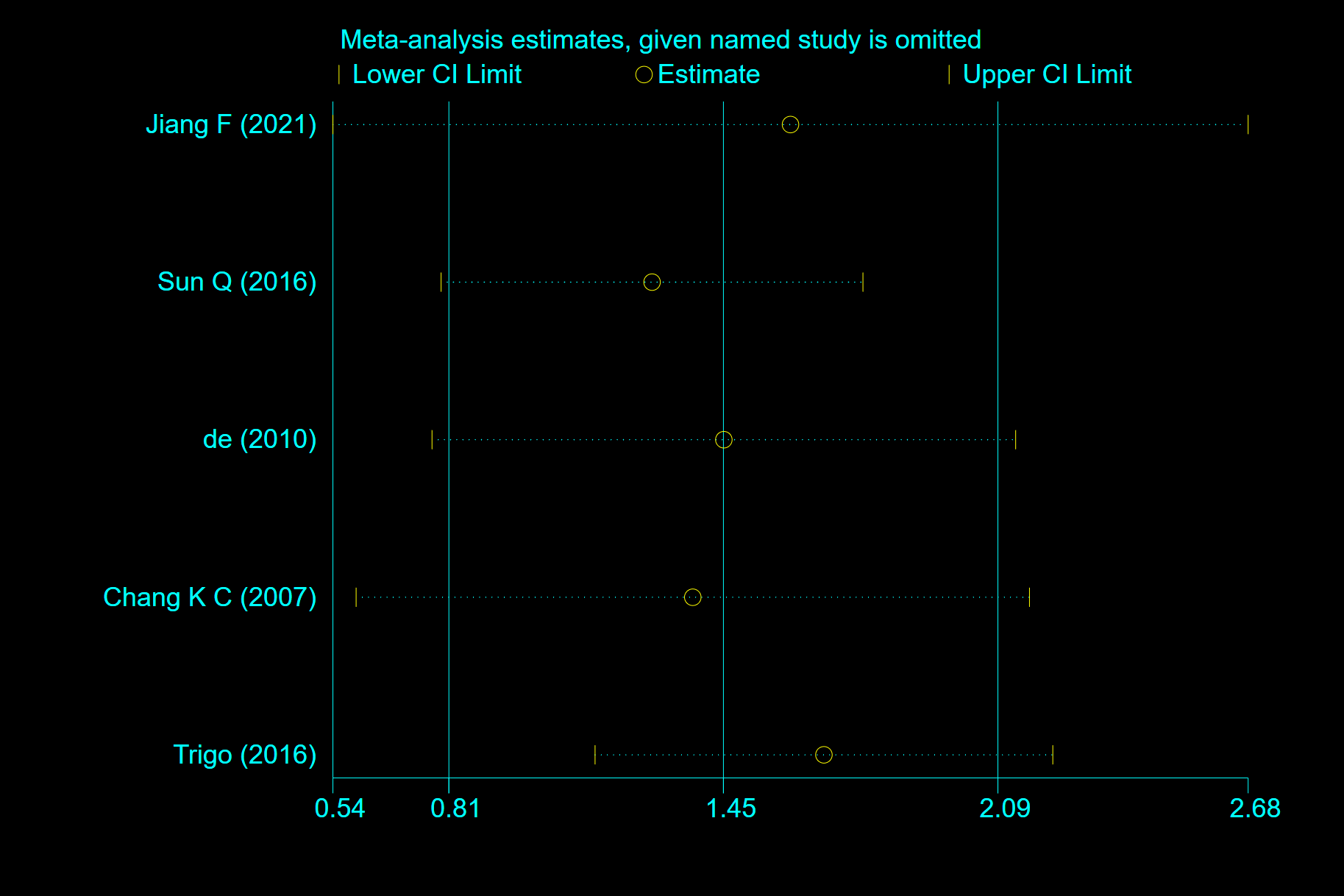
figure S4


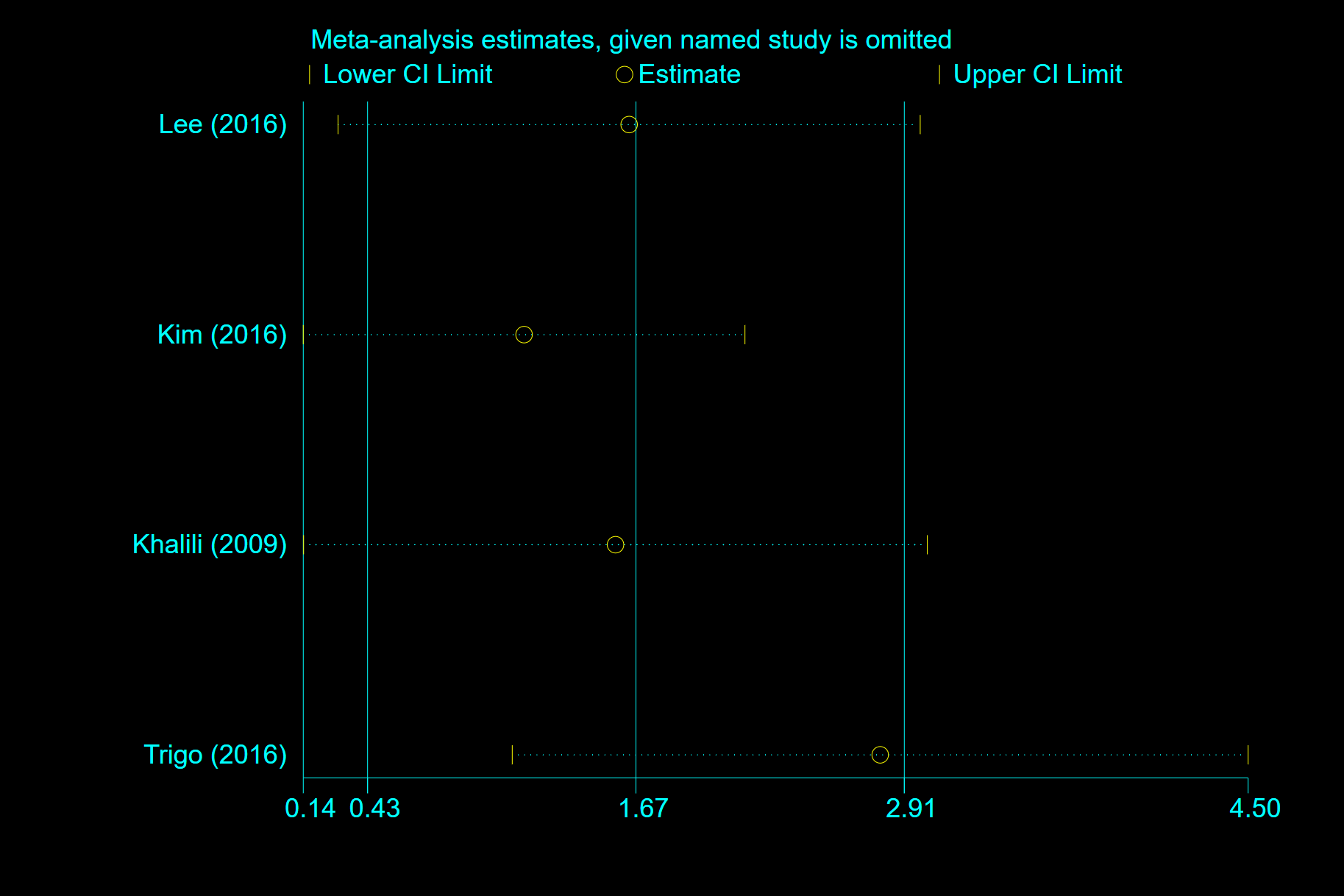
figure S5


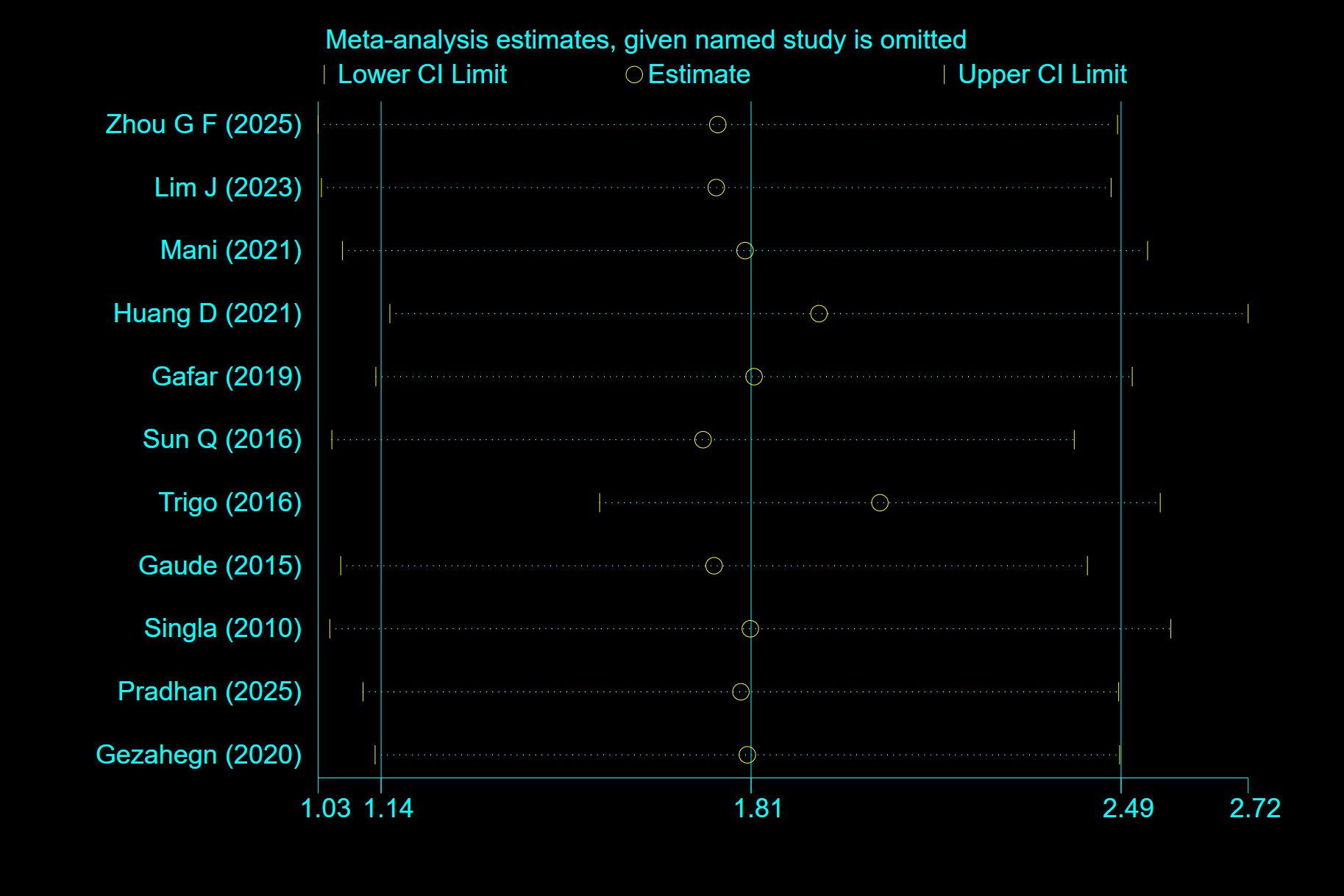
figure S6


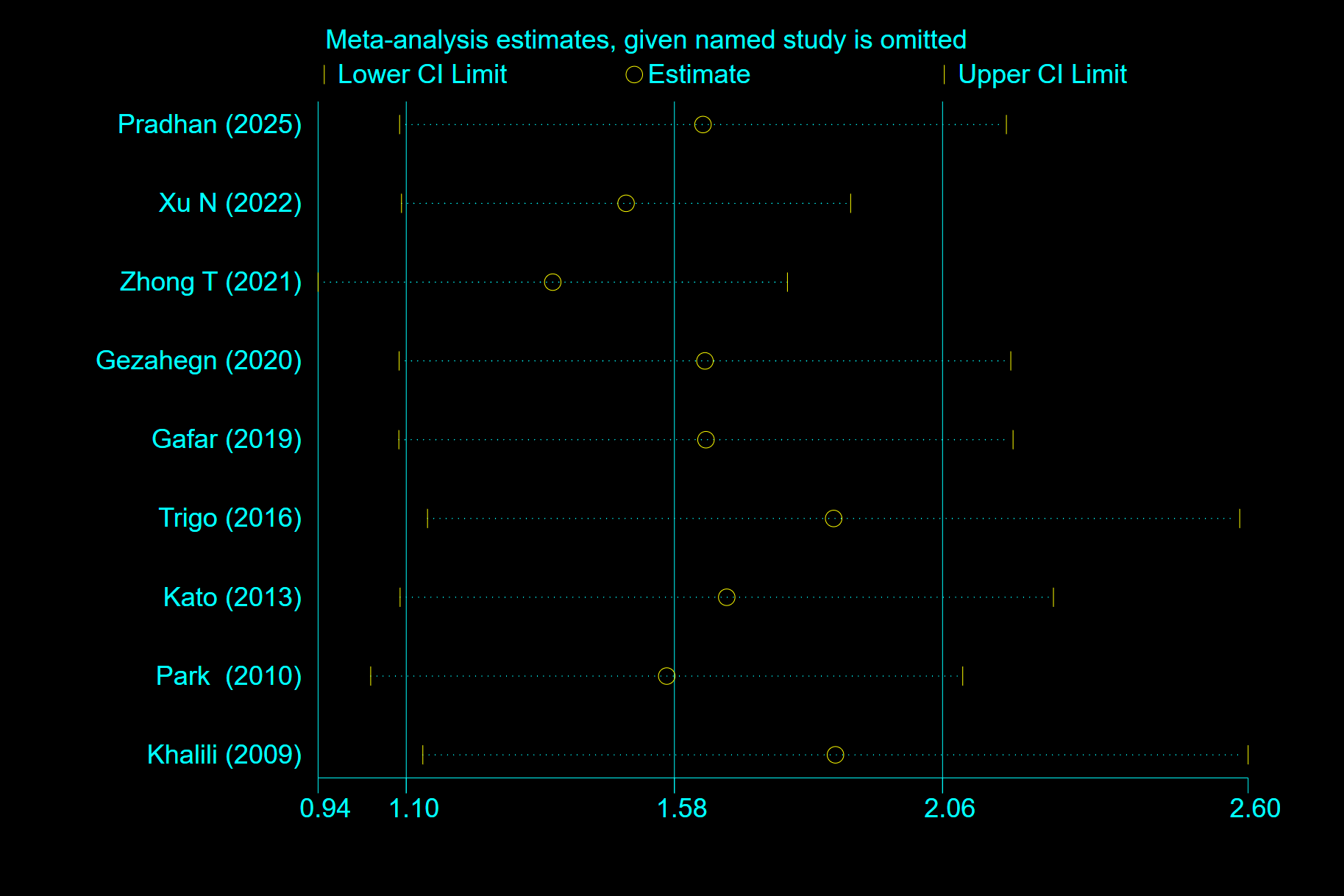
figure S7


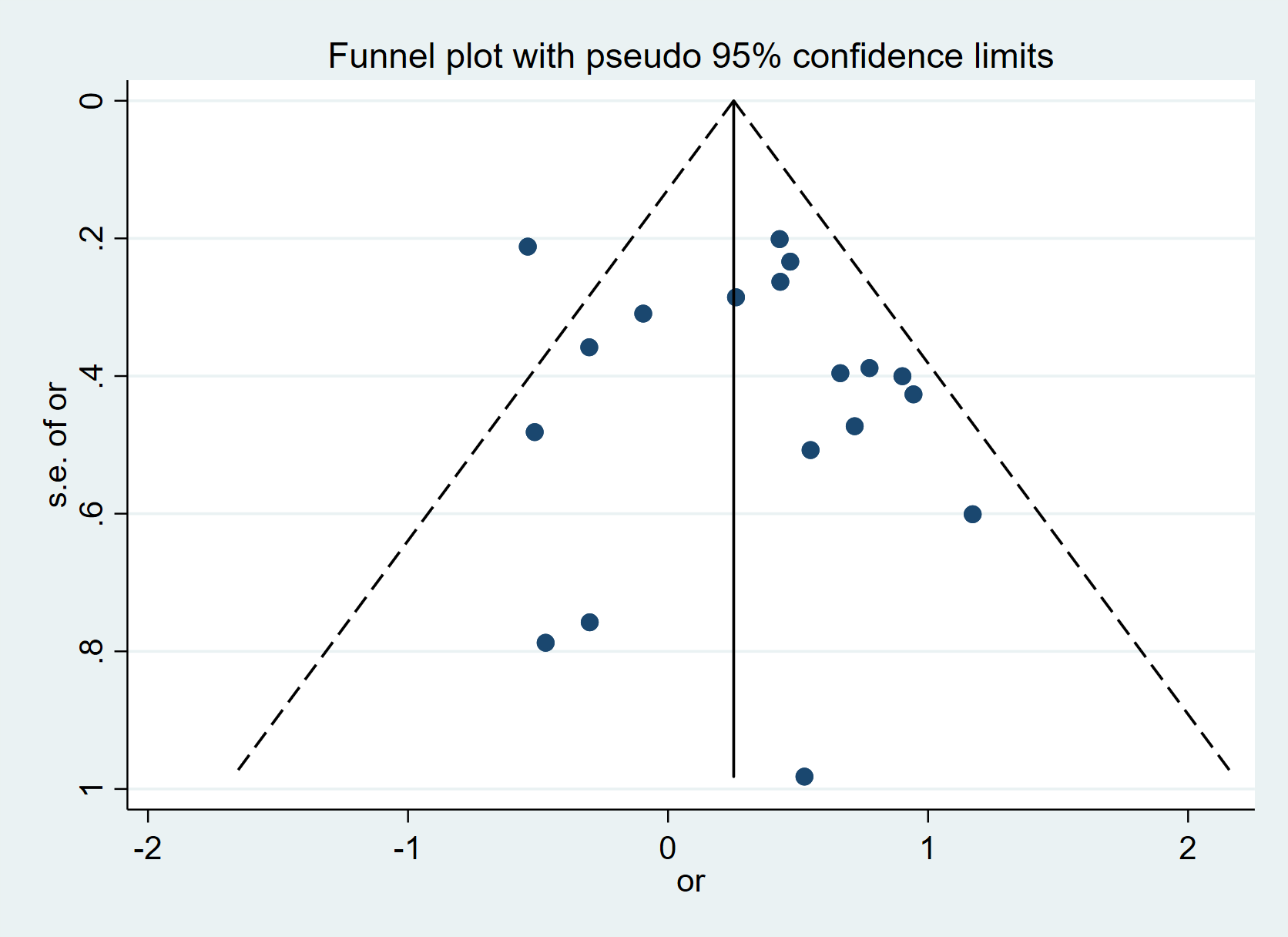
figure S8


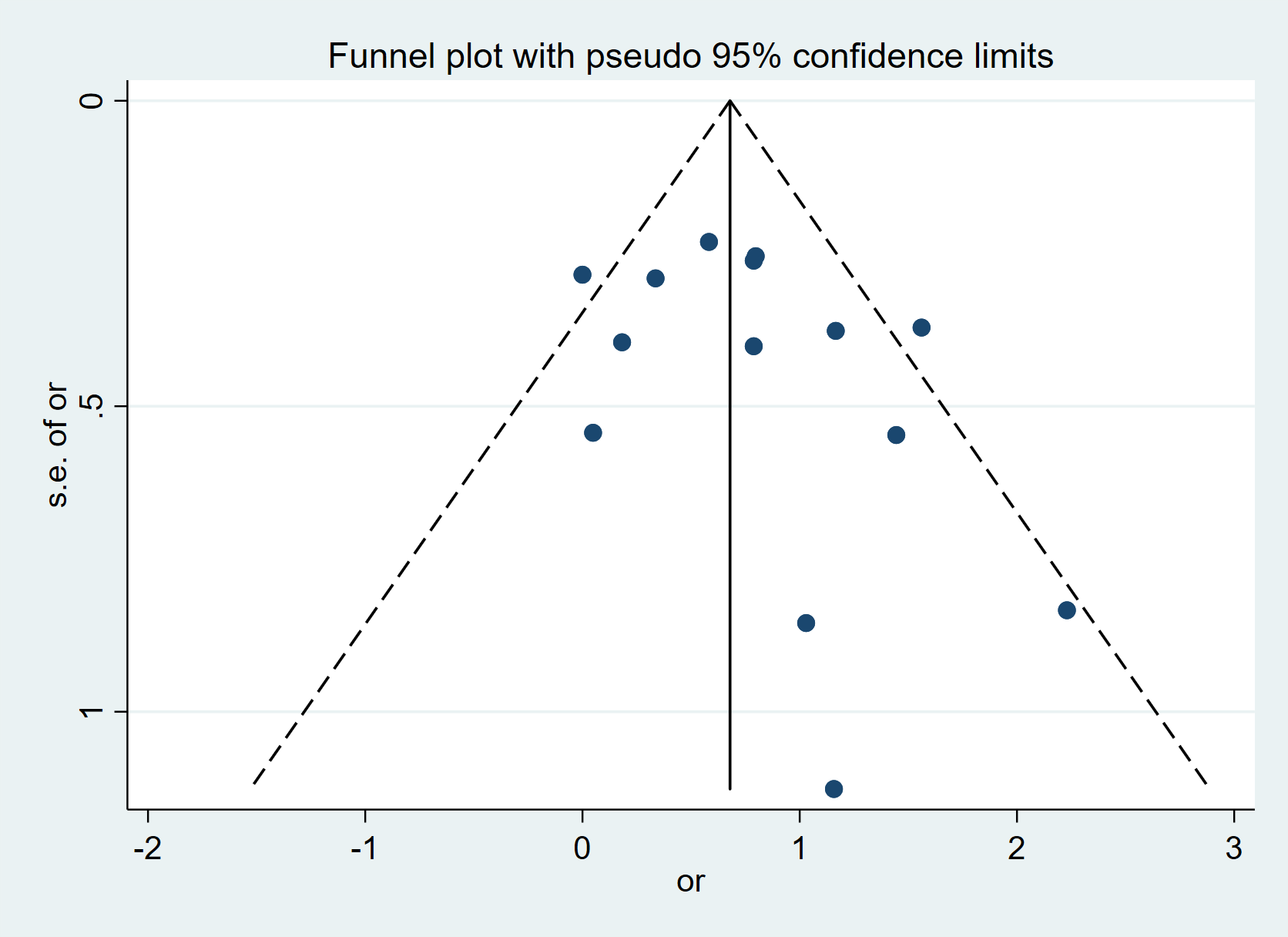
figure S9


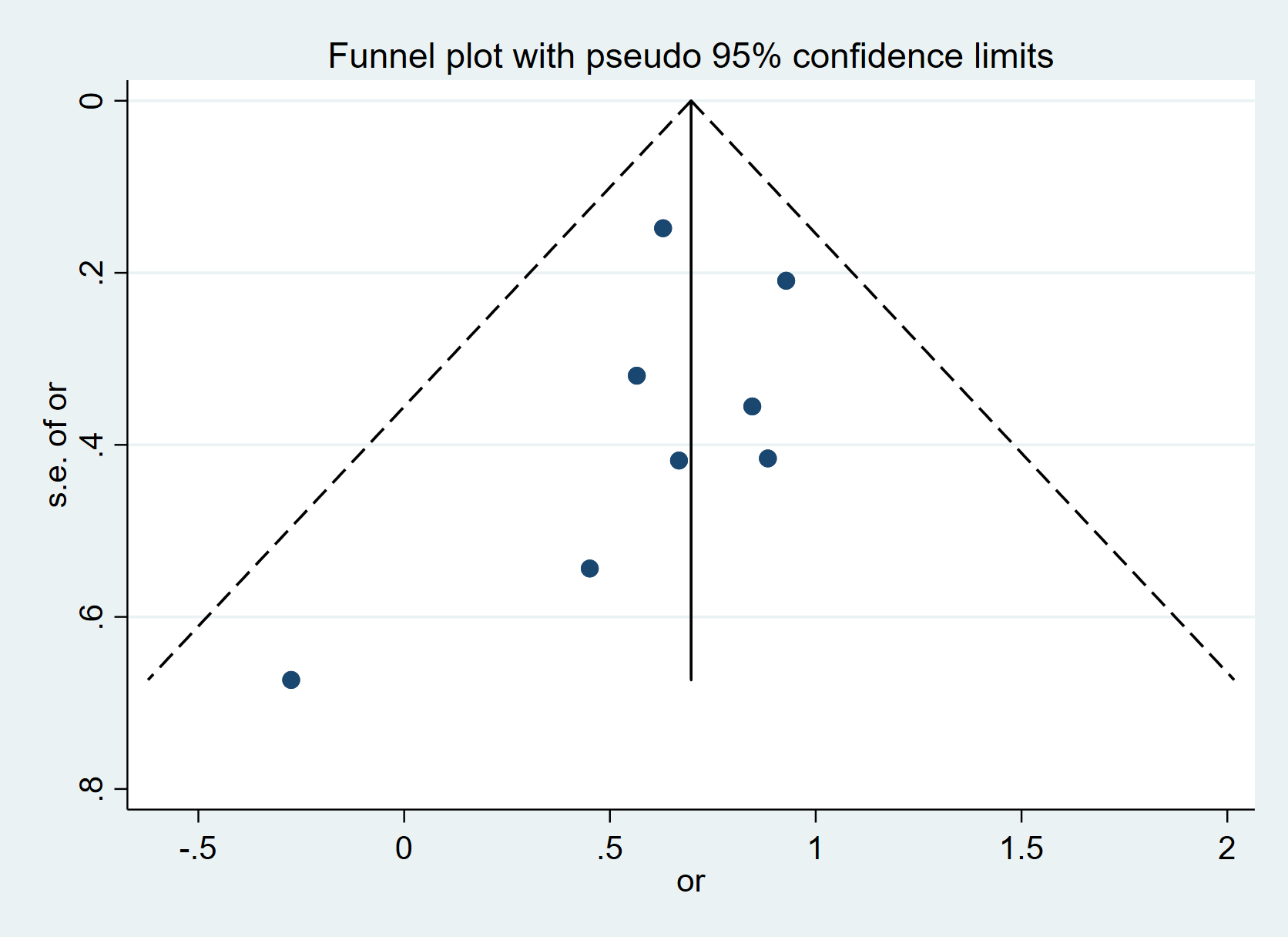
figure S10


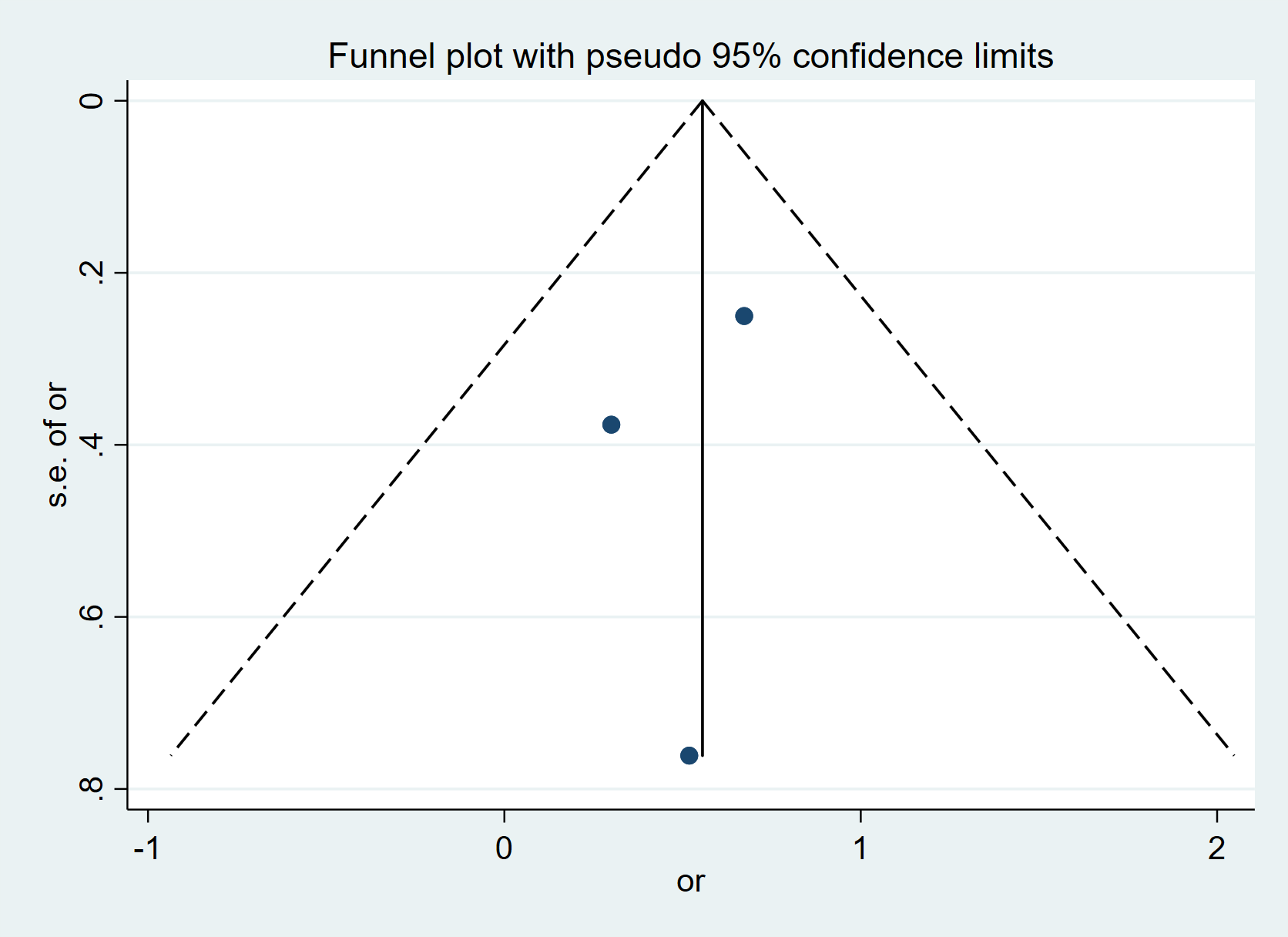
figure S11


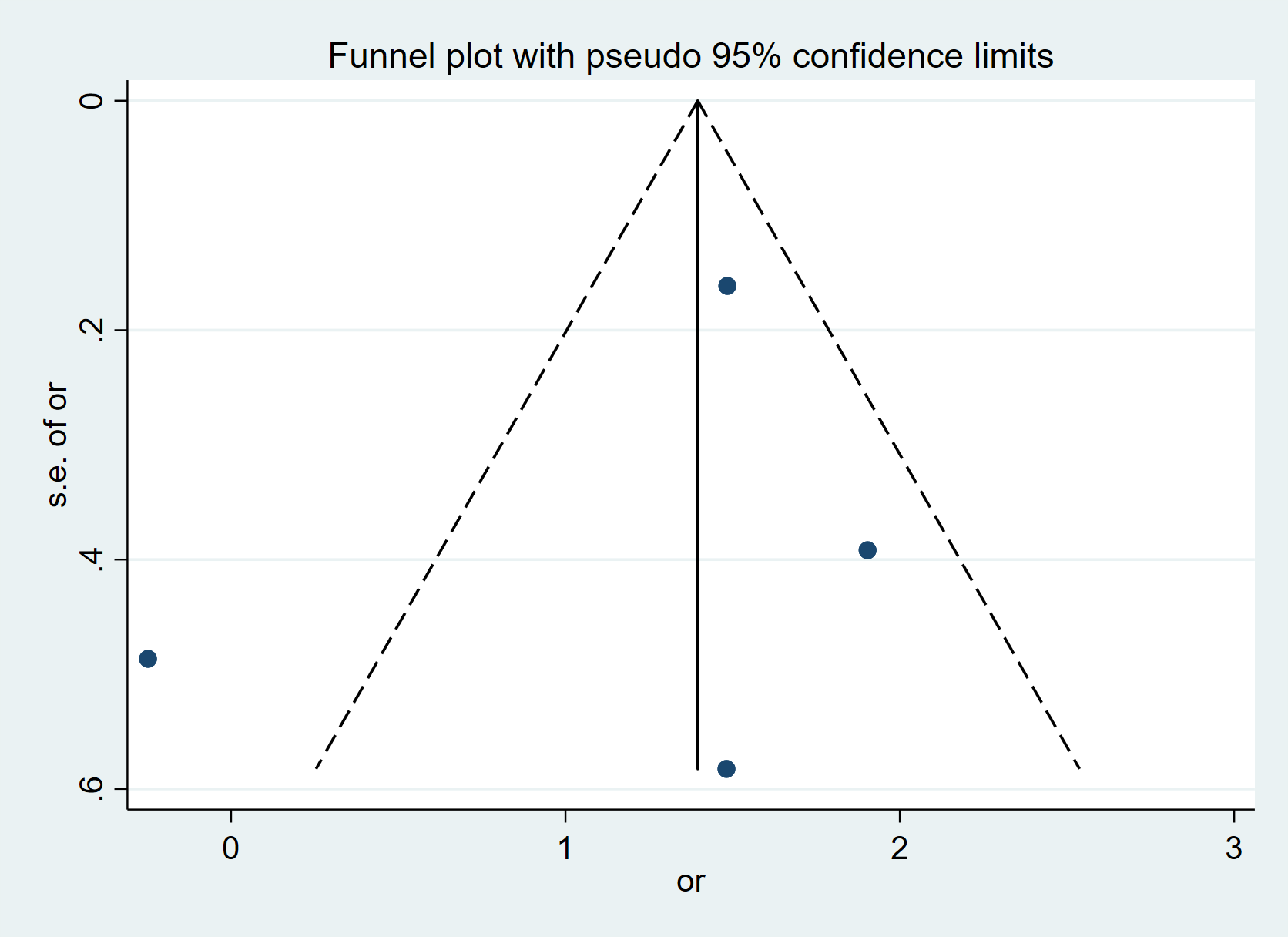
figure S12


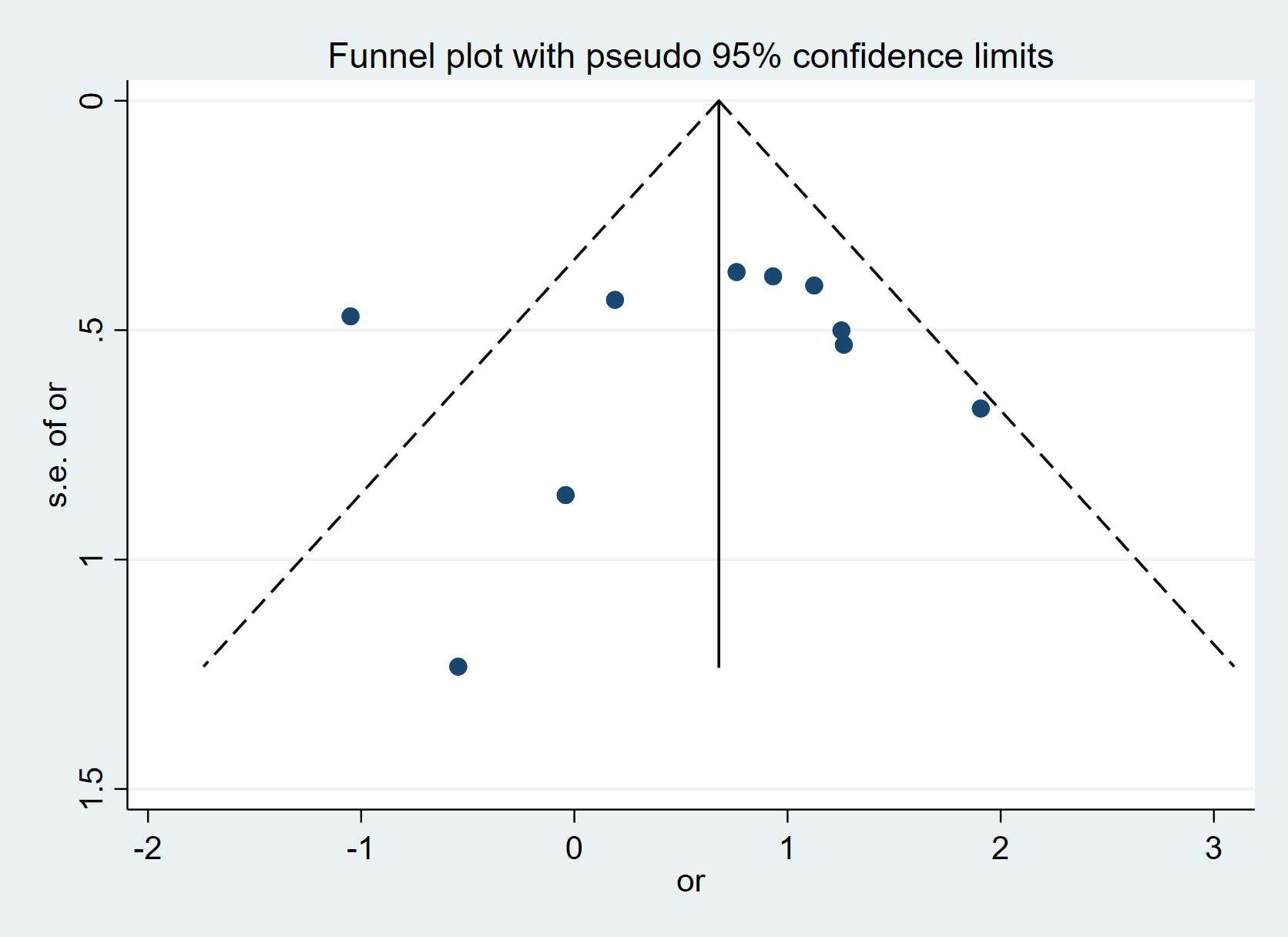
figure S13


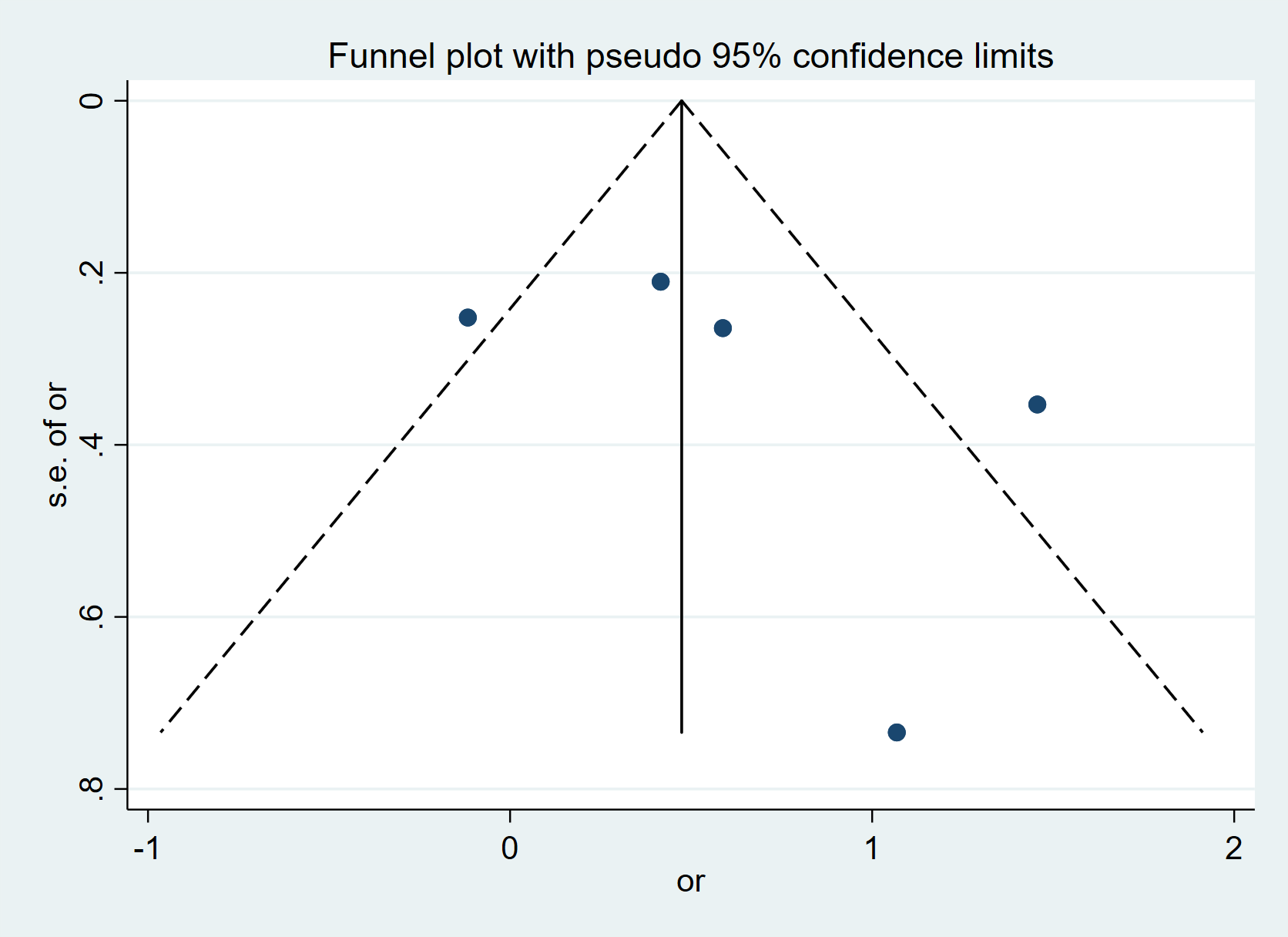
figure S14


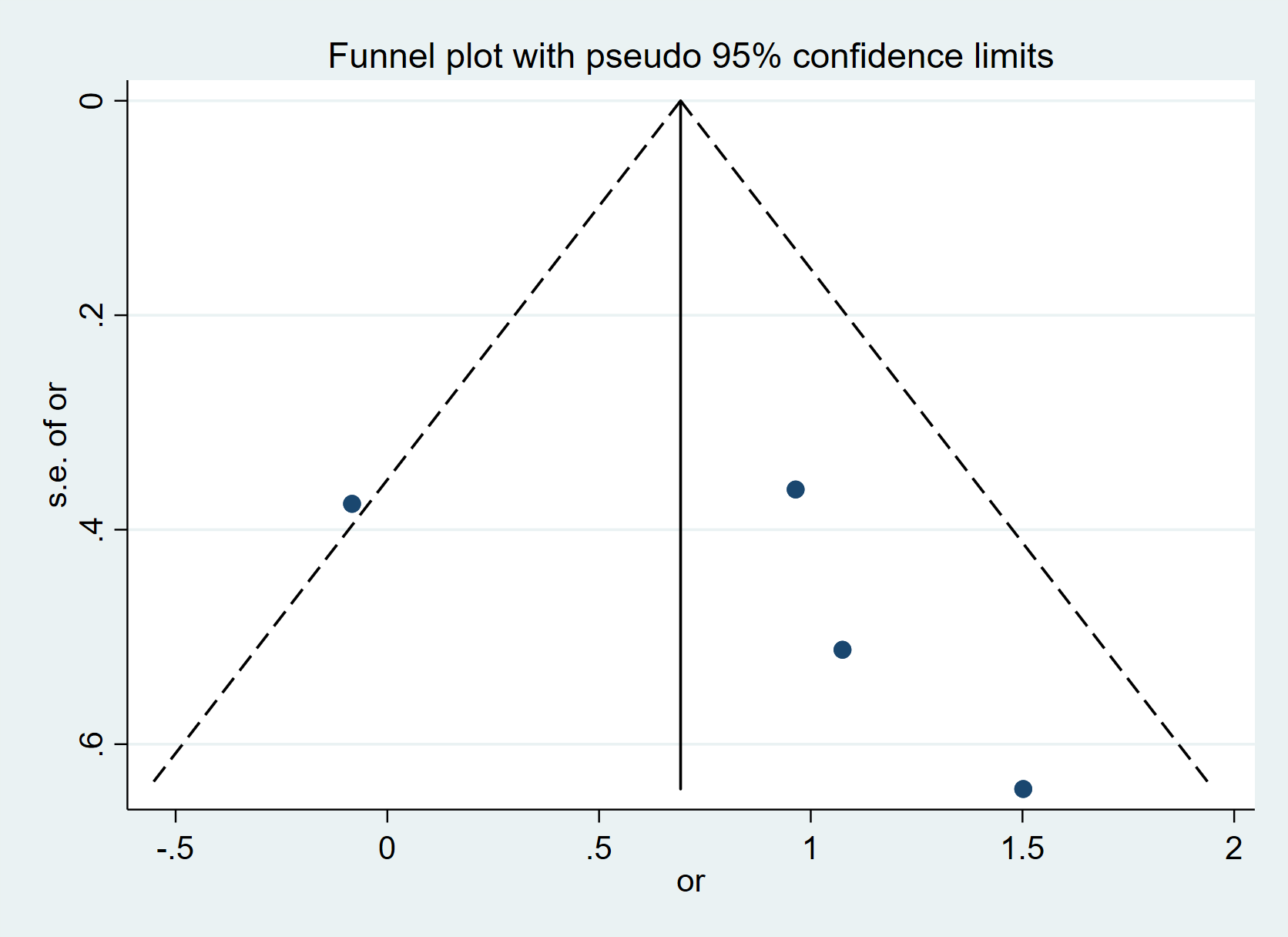
figure S15


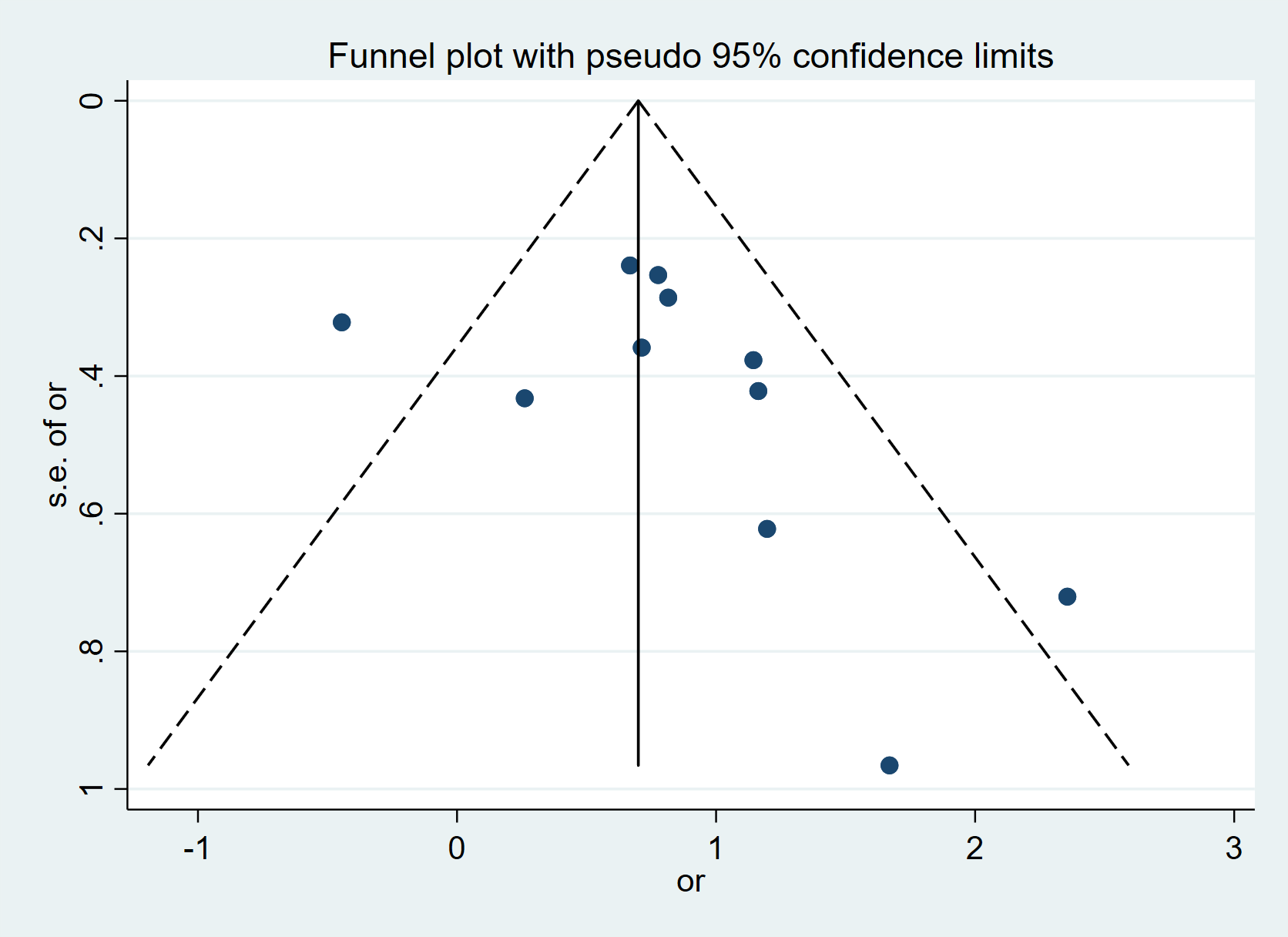
figure S16


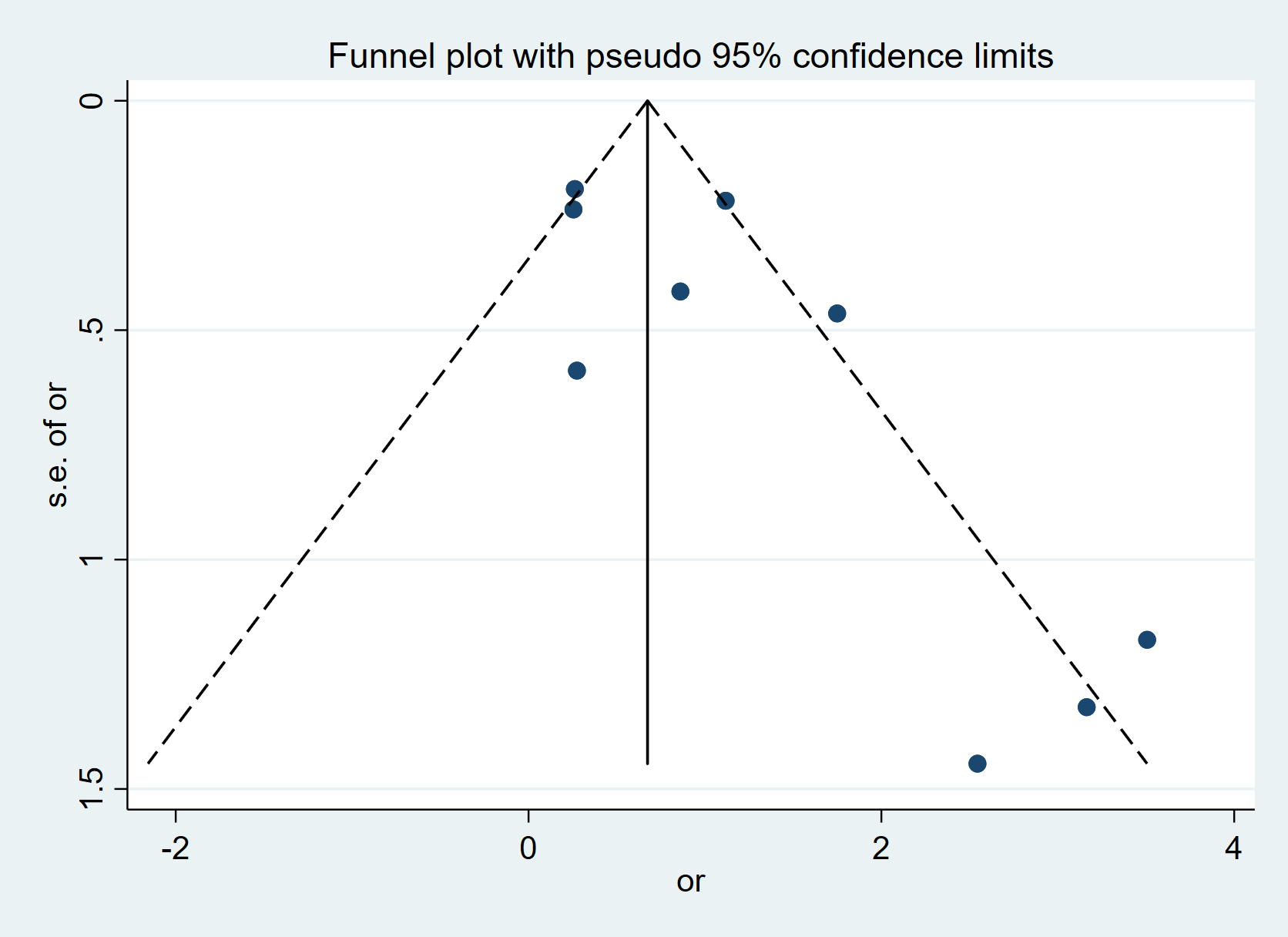
figure S17
